# New Software for the Fast Estimation of Population Recombination Rates (FastEPRR) in the Genomic Era

**DOI:** 10.1534/g3.116.028233

**Published:** 2016-03-29

**Authors:** Feng Gao, Chen Ming, Wangjie Hu, Haipeng Li

**Affiliations:** * CAS Key Laboratory of Computational Biology, CAS-MPG Partner Institute for Computational Biology, Shanghai Institutes for Biological Sciences, Chinese Academy of Sciences, Shanghai 200031, China; † University of Chinese Academy of Sciences, Beijing 100049, China

**Keywords:** population recombination rates, fast estimation, genetic map, genomic era, boosting

## Abstract

Genetic recombination is a very important evolutionary mechanism that mixes parental haplotypes and produces new raw material for organismal evolution. As a result, information on recombination rates is critical for biological research. In this paper, we introduce a new extremely fast open-source software package (FastEPRR) that uses machine learning to estimate recombination rate ρ (=$4N_{e}r$) from intraspecific DNA polymorphism data. When ρ>10 and the number of sampled diploid individuals is large enough (≥50), the variance of $\rho_{\text{FastEPRR}}$ remains slightly smaller than that of $\rho_{\text{LDhat}}$. The new estimate $\rho_{\text{comb}}$ (calculated by averaging $\rho_{\text{FastEPRR}}$ and $\rho_{\text{LDhat}}$) has the smallest variance of all cases. When estimating $\rho_{\text{FastEPRR}}$, the finite-site model was employed to analyze cases with a high rate of recurrent mutations, and an additional method is proposed to consider the effect of variable recombination rates within windows. Simulations encompassing a wide range of parameters demonstrate that different evolutionary factors, such as demography and selection, may not increase the false positive rate of recombination hotspots. Overall, accuracy of FastEPRR is similar to the well-known method, LDhat, but requires far less computation time. Genetic maps for each human population (YRI, CEU, and CHB) extracted from the 1000 Genomes OMNI data set were obtained in less than 3 d using just a single CPU core. The Pearson Pairwise correlation coefficient between the $\rho_{\text{FastEPRR}}$ and $\rho_{\text{LDhat}}$ maps is very high, ranging between 0.929 and 0.987 at a 5-Mb scale. Considering that sample sizes for these kinds of data are increasing dramatically with advances in next-generation sequencing technologies, FastEPRR (freely available at http://www.picb.ac.cn/evolgen/) is expected to become a widely used tool for establishing genetic maps and studying recombination hotspots in the population genomic era.

Genetic recombination exchanges genetic material, produces new haplotypes during meiosis, and plays a critical role in organismal evolution (Coop and Przeworski 2007). In living organisms, this process is highly regulated, and, because its rate varies along the genome, much attention has been paid to identifying recombination hotspots (Baudat *et al.* 2010). Increased knowledge about recombination will be useful for studies of linkage disequilibrium (LD) (Auton *et al.* 2013; Hill and Robertson 1968; Ohta and Kimura 1971), admixture (Price *et al.* 2009; Pugach *et al.* 2011), natural selection (Hernandez *et al.* 2011; Sattath *et al.* 2011), and associated work on genetic diseases (Weiss and Clark 2002).

Recombination rates can be estimated by experimentally counting the number of such events during meiosis (Hudson and Kaplan 1985; Myers and Griffiths 2003). However, the application of this approach is limited because of the extremely low frequency of recombination. This issue can be overcome on the one hand by sequencing a large number of parent-offspring pairs (Kong *et al.* 2010) using a large amount of sperm from a single male (Lu *et al.* 2012). On the other, the number of recombination events that occurred in the past can be inferred via coalescent theory and population genetics; in this approach, population recombination rate is denoted as $\rho =4N_{e}r$, where $N_{e}$ is the effective population size, and r the recombination rate per generation. Over the last two decades, a number of methods that use likelihood models to estimate recombination rates from intraspecific DNA polymorphism data have been proposed. Of these, full-likelihood methods, including importance sampling (Griffiths and Marjoram 1996; Fearnhead and Donnelly 2001), Markov Chain Monte Carlo (MCMC) (Kuhner *et al.* 2000), and Bayesian MCMC (Nielsen 2000; Wang and Rannala 2008, 2009) have proved the most accurate for estimating ρ. However, because full-likelihood approaches are very computationally expensive, even with moderately-sized data sets, a composite-likelihood method based on two-locus sampling probabilities was also proposed to estimate ρ (Hudson 2001). Under the infinite-site model, this method calculates the probabilities of all pairs of segregating sites, and then multiplies all these pairwise probabilities to calculate a composite likelihood. Fearnhead and Donnelly (2002) then proposed that the region of interest should be divided into subregions, with the likelihood of each subregion combined as a composite likelihood. Others have argued that, because the infinite-site model is often violated, two-locus sampling probabilities can instead be obtained using Monte Carlo simulations in a finite-site mutation model (McVean *et al.* 2002). This improved approach is implemented in the LDhat software package (http://ldhat.sourceforge.net/) with, most recently, a varying recombination rate model applied to calculate a composite likelihood (Auton and McVean 2007). Li and Stephens (2003) have also developed “product of approximate conditionals” (PAC) method, which calculates an approximation for conditional likelihood. In sum, although these composite-likelihood methods are relatively simpler computationally than full-likelihood approaches, calculations are still time-consuming.

In our recent work, building on the infinite-site model, we proposed a ρ-estimator using boosting, a machine learning method (Lin *et al.* 2013). In this context, boosting is used to select the best regression model between recombination rate and a number of summary statistics. Estimates for ρ using our new method are as precise as others, but it is biased in some circumstances. Thus it may limit the application of the machine learning method. In this paper, we extend the machine learning method and present a very fast software package (FastEPRR) to estimate population recombination rate using intraspecific DNA polymorphism data. First, because it has been suggested that it is important to consider the finite-site model when estimating the recombination rate (McVean *et al.* 2002), our implementations take into account violations of the infinite-site model (*i.e.*, multiple hits). Second, we introduce a linear correction and demonstrate that estimates using FastEPRR are unbiased. Third, we propose a method (Supplemental Material, Figure S1) to take into account the effects of variable recombination rates within windows. Finally, as a test case, we analyze the 1000 Genomes phased OMNI data set (Altshuler *et al.* 2012) to calculate local recombination rates for three major human populations with African (YRI), European (CEU), and East Asian (CHB) ancestry. The Pearson correlation coefficient between estimates made using either FastEPRR or LDhat is very high, and ranges between 0.929 and 0.987 at a 5-Mb scale. Notably, to estimate the genome-wide recombination rates for one population, FastEPRR only needs less than 3 d based on a single CPU core of a computer. Indeed, when a computer cluster was used, the analysis was completed in just a few hours; therefore, use of FastEPRR dramatically reduces the time required to estimate genome-wide recombination rates, and is just as precise as the well-known method, LDhat.

## Materials and Methods

### Summary statistics

Demography and selection affect the mutation frequency spectrum (Figure S2), especially the frequency of singletons (Fu and Li 1993). However, we would not suggest simulating data conditional to the mutation frequency spectrum since the importance sampling is relatively time consuming. Instead, we use the compact folded mutation frequency spectrum, named by Li and Stephan (2005), to partially quantify the effects of demography and selection. Indeed, this approach might improve the accuracy of estimates under certain conditions. Suppose that the number of chromosomes (n) in a sample is ≥6, and ${\text{ξ}}_{i}$ is the number of derived mutations that occur on i chromosomes; in this case, the compact folded mutation frequency spectrum is denoted $\left\{\xi_{1}^{\text{′}},\;\;{\text{ξ}}_{2}^{\text{′}},\;{\text{ ξ}}_{x}^{\prime }\right\}$, where ${\text{ξ}}_{1}^{\text{′}}={\text{ξ}}_{1}+{\text{ξ}}_{n-1}$, ${\text{ξ}}_{2}^{\text{′}}={\text{ξ}}_{2}+{\text{ξ}}_{n-2}$, and ${\text{ξ}}_{x}^{\text{′}}=\overset{n-3}{\underset{i=3}{\sum }}{\text{ξ}}_{i}$. Because the number of folded singletons (${\text{ξ}}_{1}^{\text{′}}$) will impart little information about recombination, these can be excluded for analysis. The folded singletons are the derived mutations that occur on one and (n−1) chromosomes.

Let SS denote the four summary statistics; the mean value of $S_{k}^{2}$ (Hudson 1987) and $r^{2}$ (Hudson 1985) for all SNP pairs, haplotype heterozygosity, and the number of different haplotypes (H). We implemented these four summary statistics in FastEPRR because they contain considerable information about recombination (Wall 2000; Li and Stephens 2003; Kong *et al.* 2008; Lin *et al.* 2013), and excluded the folded singletons to calculate SS.

### Regression and linear correction

To obtain the regression model of population recombination rate and the summary statistics conditional on ${\text{ξ}}_{2}^{\text{′}}$ and ${\text{ξ}}_{x}^{\text{′}}$, we first generated a training set using ρ=0, 0.5, 1, 2, 5, 10, 20, 40, 70, 110, and 170. This training set was simulated using our modified Hudson’s *ms* simulator (Hudson 2002), conditional on ${\text{ξ}}_{2}^{\text{′}}$, ${\text{ξ}}_{x}^{\text{′}}$ and ρ (and the pattern of missing data, if necessary), with 100 replicates. Then we used the gamboost (Hothorn *et al.* 2015) to fit the training set and to establish the regression model ρ=f(SS). Given the observed four summary statistics (${SS}_{\text{obs}}$), recombination rate was estimated to be $\hat{\rho }=f\left({SS}_{\text{obs}}\right)$.

We next performed a linear correction to obtain an unbiased estimate by generating 100 simulated data sets given $\hat{\rho }$ and estimated ${\hat{\rho }}_{s}$ for each. In this case, we use $\text{mean (}{\hat{\rho }}_{s})$ as the mean value for estimated ${\hat{\rho }}_{s}$ in the simulated data sets, and $\text{α}=\hat{\rho }\text{/mean(}{\hat{\rho }}_{s})$. Thus, estimated recombination rate is $\text{α}\hat{\rho }$ following linear correction.

It is worth noting that gamboost could produce biased estimates if the real ρ falls out of the range of the training ρ (Lin *et al.* 2013). More accurately, the observed number of different haplotypes ($H_{\text{obs}}$) should fall within the range of H in the training set. If we let $H_{\text{thres}}$ be the 95th percentile for H given ρ=170, if $H_{\text{obs}}>H_{\text{thres}}$, and extend the range of the training ρ (*i.e.*, ρ= 0, 0.5, 1, 2, 5, 10, 20, 40, 70, 110, 170, 180, 190, 200, 220, 250, 300, and 350) a new regression model can be obtained and the recombination rate re-estimated.

### Variable recombination rates within windows

When estimating recombination rate for a given window, we assume that this is constant. However, because this may not be correct, the effect of a variable recombination rate within a given window can be investigated by sliding others over it. For example, if we consider four overlapping sliding windows (*i.e.*, win1, win2, win3, and win4) each with a step length half their size (Figure S1), we can denote ${\hat{\rho }}_{1}$, ${\hat{\rho }}_{2}$, ${\hat{\rho }}_{3}$ and ${\hat{\rho }}_{4}$ as the estimated recombination rate, respectively. If $\rho_{1}$, $\rho_{2}$, and $\rho_{3}$ are the real recombination rates of these windows, then we have $\rho_{1}=x_{1}+x_{2}$, $\rho_{2}=x_{2}+x_{3}$, $\rho_{3}=x_{3}+x_{4}$, where $x_{i}$ denotes the recombination rate for the *i*-th region. To estimate $x_{1}$, $x_{2}$, $x_{3}$, and $x_{4}$, three constraint conditions can be introduced in order:

(1) $x_{1}\geq 0,\;x_{2}\geq 0,\;x_{3}\geq 0,\;\text{and }x_{4}\geq 0$.
(2) Minimize $f_{1}={\left(x_{1}+x_{2}-{\hat{\rho }}_{1}\right)}^{2}+{\left(x_{2}+x_{3}-{\hat{\rho }}_{2}\right)}^{2}+{\left(x_{3}+x_{4}-{\hat{\rho }}_{3}\right)}^{2}$.
(3) Maximize $f_{2}=x_{1}x_{2}x_{3}x_{4}$.

Of these, the first condition is easy to accept because recombination rate should be positive, and since ${\hat{\rho }}_{i}$ is the observed value, and $x_{i}$ the predicted, the second condition ($f_{1}$) denotes the total error in prediction. Our objective is to minimize $f_{1}$ using the least squares principle, and because we also aim to maximize Shannon entropy in information theory (Shannon 1948), we include the third condition. The detailed solution is provided in File S1.

Note that, conditional on ${\hat{\rho }}_{1}$, ${\hat{\rho }}_{2}$, and ${\hat{\rho }}_{3}$, using this procedure $x_{1}$, $x_{2}$, $x_{3}$, and $x_{4}$ can be estimated, and that conditional on ${\hat{\rho }}_{2}$, ${\hat{\rho }}_{3}$, and ${\hat{\rho }}_{4}$, $x_{2}$, $x_{3}$, and $x_{4}$ can be re-estimated. Thus, the estimated ${\hat{x}}_{i}$ is the mean value of all predicted values for $x_{i}$.

### Validating FastEPRR using simulated data

To validate the performance of FastEPRR, we compared it to our earlier regression-based method (gam), as well as to the composite-likelihood method (implemented in LDhat). Estimates from these three methods are denoted $\rho_{\text{FastEPRR}}$, $\rho_{\text{gam}}$, and $\rho_{\text{LDhat}}$, respectively. In order to estimate $\rho_{\text{gam}}$, we used a nonparametric model (*i.e.*, a generalized additive model) based on H for training (ρ= 20, 60, 100, 140, and 180) following Lin *et al.* (2013).

To estimate $\rho_{\text{LDhat}}$, we first used the *complete* program to calculate the likelihoods of all two-locus haplotype configurations, with a population mutation rate θ=0.01 and the maximum ρ=300. Second, we used the *pairwise* program to estimate the recombination rate.

We studied cases with different sample sizes (*i.e.*, n=50, 100, and 200), where n is the number of chromosomes but did not include larger sample sizes because LDhat computing time increases dramatically in these cases. Results were not achieved even when we ran LDhat on a state-of-the-art computer cluster with more than 1000 computing nodes.

We simulated neutral data using the coalescent simulator Hudson’s *ms*, while the data set considered with the hitchhiking model (*i.e.*, positive selection) was simulated using *msms* (Ewing and Hermisson 2010). To assess the impact of missing data, we treated *ms* output as a two-dimensional array (*i.e.*, sampled chromosomes as rows and polymorphic sites as columns), and randomly selected v% cells and marked them as question marks (to denote missing data). In this part of the study, we examined cases of  v= 1, 5, 20, and 30.

To investigate potential bias due to the phasing process, we randomly paired simulated haplotypes to form genotypes (*i.e.*, n haplotypes to n/2 genotypes). These haplotypes were then reinferred using PHASE v2.1.1 (Stephens *et al.* 2001; Stephens and Scheet 2005) based on their genotypes and recombination rate estimated from inferred haplotypes.

### Application of FastEPRR

To test the application of FastEPRR, we used it to analyze the 1000 Genomes phased OMNI data set (Altshuler *et al.* 2012). We selected three major human populations for this analysis: 88 individuals from Yoruba in Ibadan, Nigeria (YRI); 85 Utah resident individuals with northern and western European ancestry (CEU); and 97 Han Chinese individuals from Beijing, China (CHB). To estimate local recombination rates on the 22 autosomes, we first scanned each chromosome with nonoverlapping 50 kb sliding windows. For this step, ${\text{ξ}}_{2}^{\text{′}}$ and ${\text{ξ}}_{x}^{\text{′}}$, the four summary statistics, and the start and end positions of the windows were stored in order as files. Indels and polymorphic sites were excluded from the analysis if their quality score was less than 20, and windows were excluded if they overlapped with known gaps in the reference genome sequence, or if their number of segregating sites (${\text{ξ}}_{2}^{\text{′}}+{\text{ξ}}_{x}^{\text{′}}$) was less than 10. Next, we obtained regression models for each unique combination of ${\text{ξ}}_{2}^{\text{′}}$ and ${\text{ξ}}_{x}^{\text{′}}$, and then applied these to estimate recombination rates in windows that had the same combination of ${\text{ξ}}_{2}^{\text{′}}$ and ${\text{ξ}}_{x}^{\text{′}}$ for all autosomes. Finally, we merged the recombination rates for all windows to calculate a rate for each autosome, and repeated the analysis for the YRI, CEU, and CHB data sets.

In order to convert estimated ρ into r, we first estimated $N_{e}$ by comparing the total length of the $\rho_{\text{FastEPRR}}$ map with overlapping sections of the 2010 deCODE family-based map (Kong *et al.* 2010). This map provides per generation recombination rates at a 10-kb scale. To obtain pairwise Pearson correlation coefficients for the $\rho_{\text{FastEPRR}}$ map, the $\rho_{\text{LDhat}}$ map (Altshuler *et al.* 2012), and the 2010 deCODE map for different populations, the three were compared to one another at 50-kb and 5-Mb scales.

To consider the effects of variable recombination rates within windows, we scanned each autosome with overlapping sliding windows (*i.e.*, window size, 50 kb and step length, 25 kb). Following the method described above (Figure S1), we then obtained a genetic map at the 25-kb scale, finer than that at the 50-kb scale.

### Implementation

FastEPRR is an R package (open source) that can run across a range of platforms once a standard environment has been installed. This software can be downloaded from our institutional website (http://www.picb.ac.cn/evolgen/softwares/) along with the related genetic maps.

### Data availability

The authors state that all data necessary for confirming the conclusions presented in the article are represented fully within the article.

## Results

**Figure 1:**
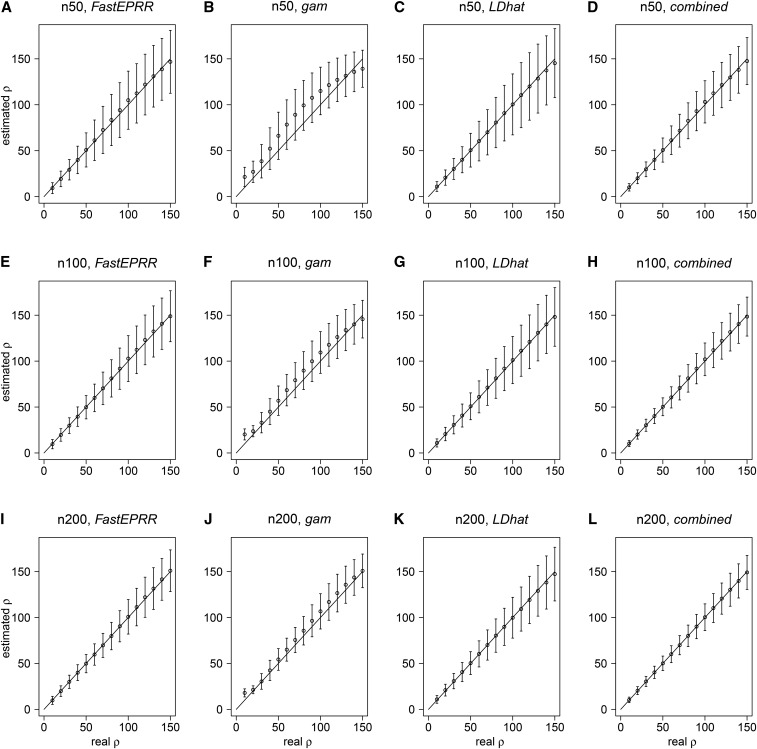
Comparison of $\rho_{\text{FastEPRR}}$, $\rho_{\text{gam}}$, $\rho_{\text{LDhat}}$, and $\rho_{\text{comb}}$. We compared $\rho_{\text{FastEPRR}}$ (A), (E), and (I) with $\rho_{\text{gam}}$ (B), (F), and (J), $\rho_{\text{LDhat}}$ (C), (G), and (K), and $\rho_{\text{comb}}$ (D), (H), and (L), with the sample sizes of n=50 (A)–(D), 100 (E)–(H) and 200 (I)–(L). The number of segregating sites S=45 (when n=50), 52 (when n=100), and 59 (when n=200). The mean and the SD of $\hat{\rho }$ were estimated using $10^{4}$ simulated data conditional on ρ and S, unless noted otherwise.

It has been shown that $\rho_{\text{gam}}$ is biased when sample size is small (Lin *et al.* 2013). We examined the accuracy of $\rho_{\text{FastEPRR}}$ by comparing it to $\rho_{\text{gam}}$ and $\rho_{\text{LDhat}}$ with a fixed number of segregating sites. Results show that $\rho_{\text{FastEPRR}}$ is an improvement on $\rho_{\text{gam}}$, and remains unbiased in the cases we examined (Figure 1), as a linear correction is implemented by FastEPRR. When sample size is small (n=50), $\rho_{\text{FastEPRR}}$ has the same level of accuracy as $\rho_{\text{LDhat}}$ in mean, standard deviation, and the root mean square error (RMSE), while $\rho_{\text{gam}}$ produces estimates with fairly small SD but a certain bias (Figure 1, A–C). When sample size is larger (n≥100), the accuracy level of the three methods is almost the same (Figure 1, E–G, and I–K). When it is very large (n=1000), FastEPRR still performs well. Indeed, in these cases, the SD of $\rho_{\text{FastEPRR}}$ is smaller than that seen in small sample size examples (Figure 1, A, E, and I, and Figure S3). We further investigated this issue and observed that the RMSE of $\rho_{\text{FastEPRR}}$ gradually decreases as sample size increases (Table S1), which suggests that the accuracy of $\rho_{\text{FastEPRR}}$ is improved at larger sample sizes.

We then examined the correlation between $\rho_{\text{FastEPRR}}$ and $\rho_{\text{LDhat}}$ in our three simulated cases (Figure 1), and show that the Pearson correlation coefficient is 0.717 (n=50), 0.804 (n=100), and 0.852 (n=200), respectively. The Pearson correlation coefficient is less than 1 because $\rho_{\text{FastEPRR}}$ and $\rho_{\text{LDhat}}$ are based on different recombination signatures. Thus, to improve the accuracy of the estimated recombination rate, we propose a new estimate that combines $\rho_{\text{FastEPRR}}$ and $\rho_{\text{LDhat}}$ together. In this estimate, we denote $\rho_{\text{comb}}=(\rho_{\text{FastEPRR}}+\rho_{\text{LDhat}})/2$; because both $\rho_{\text{FastEPRR}}$ and $\rho_{\text{LDhat}}$ are unbiased, $\rho_{\text{comb}}$ will be also unbiased (Figure 1, D, H, and L). Indeed, in this case, the SD and RMSE of $\rho_{\text{comb}}$ are smallest (Table S2), indicating that $\rho_{\text{comb}}$ is the most accurate way to estimate recombination rate. Similarly, when $\text{θ}\;(=4N_{e}\mu )$ is fixed, $\rho_{\text{FastEPRR}}$ has the same accuracy as $\rho_{\text{LDhat}}$ (n=100, θ=10), and the SD of $\rho_{\text{comb}}$ is smallest (Table 1).

**Table 1:** Comparison of $\rho_{FastEPRR}$, *$\rho_{\text{LDhat}}$* and $\rho_{comb}$ when θ(=4Nμ) is fixed

Real ρ	FastEPRR	LDhat	Combined	Real ρ	FastEPRR	LDhat	Combined
10	9.4 (5.2)	10.8 (4.3)	10.1 (3.9)	90	91.5 (23.0)	91.4 (24.1)	91.4 (19.9)
20	19.8 (6.9)	20.7 (7.5)	20.2 (6.0)	100	102.5 (25.0)	101.4 (26.3)	101.9 (21.7)
30	29.8 (8.7)	31 (10.1)	30.4 (7.9)	110	113.3 (26.6)	111.9 (28.0)	112.6 (23.0)
40	39.4 (10.7)	40.7 (12.5)	40.0 (9.9)	120	123.1 (27.1)	121.3 (29.8)	122.2 (24.1)
50	49.6 (12.8)	50.9 (15.1)	50.2 (11.8)	130	132.4 (27.8)	131.0 (31.0)	131.7 (24.9)
60	60.0 (15.3)	61.0 (17.3)	60.5 (13.8)	140	140.8 (28.0)	139.6 (31.9)	140.2 (25.2)
80	81.5 (20.8)	81.7 (22.0)	81.6 (18.2)	150	148.7 (28.3)	148.4 (31.7)	148.5 (25.4)

n=100 and θ=10. SD of $\hat{\rho }$ shown in brackets.

**Figure 2:**
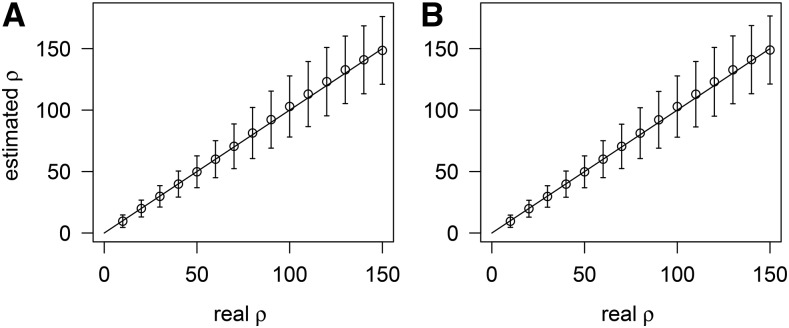
Comparison of $\rho_{\text{FastEPRR}}$ with (A), and without (B), multiple hits when n=100. When 52 mutations occur randomly on a 150 bp fragment (A), the probability of multiple hits is 0.99, but when 52 mutations occur randomly on a 10,000 bp fragment (B), this probability decreases to 0.12.

Because the rate of multiple hits is high in many viruses and bacteria (McVean *et al.* 2002), we examined the sensitivity of $\rho_{\text{FastEPRR}}$ to multiple hits under the finite-site model, and found that $\rho_{\text{FastEPRR}}$ remains unbiased (Figure 2). In the same way that LDhat considers only sites with two alleles, FastEPRR examines sites where two or more alleles are segregated.

**Figure 3:**
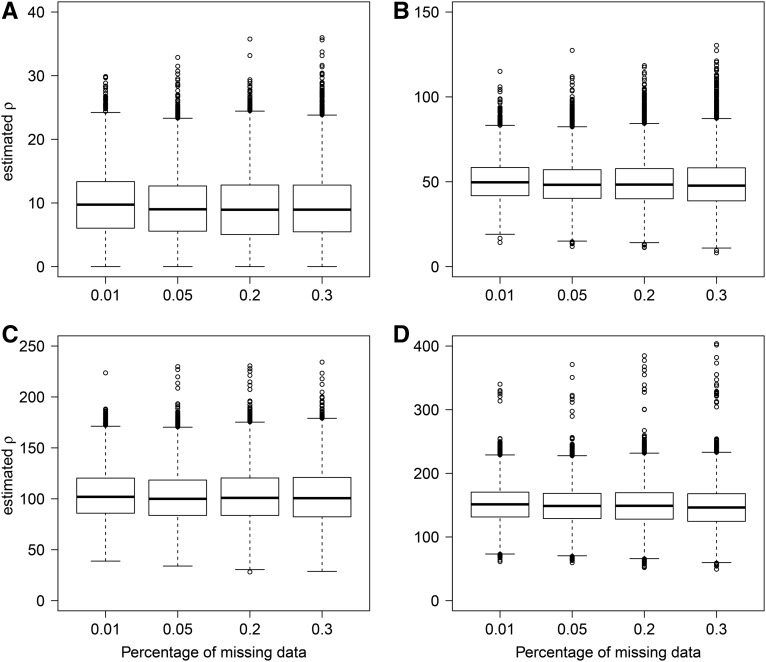
Comparisons of $\rho_{\text{FastEPRR}}$ including missing data when n=100, S=52. The real ρ= 10 (A), 50 (B), 100 (C), and 150 (D).

We also considered cases with missing data because it is often expected in sets of genome-wide DNA polymorphisms, especially when sequencing coverage is low. As FastEPRR relies on machine learning, a training set can be generated that has the same pattern of missing data as the input, and $\rho_{\text{FastEPRR}}$ can be estimated. When we did this, we found that the SD of $\rho_{\text{FastEPRR}}$ increases slightly as the percentage of missing data rises (Figure 3). Nevertheless, $\rho_{\text{FastEPRR}}$ still provides a precise and unbiased estimate even when the percentage of missing data are very high (30%).

Because FastEPRR requires haplotype information, phased intraspecific DNA polymorphism data has to be used to estimate recombination rate. To study the effect of phasing uncertainty, we compared $\rho_{\text{phasedHap}}$ (estimated from phased haplotypes) and $\rho_{\text{realHap}}$ (estimated from real haplotypes). We found that $\rho_{\text{phasedHap}}$ remained unbiased in all the cases we examined (Table 2) and, as expected, the SD of $\rho_{\text{phasedHap}}$ is slightly larger than that of $\rho_{\text{realHap}}$ when the recombination rate is large (ρ≥100).

**Table 2:** Comparing the performance of FastEPRR when information on real haplotypes is available ($\rho_{\text{realHap}}$), and when the inferred haplotypes are used ($\rho_{\text{phasedHap}}$)

Real ρ	**${\text{ρ}}_{\text{realHap}}$**	**${\text{ρ}}_{\text{phasedHap}}$**	Real ρ	**${\text{ρ}}_{\text{realHap}}$**	**${\text{ρ}}_{\text{phasedHap}}$**
10	9.6 (5.0)	9.7 (5.0)	90	91.7 (22.3)	90.1 (22.3)
20	19.6 (6.8)	19.5 (6.8)	100	102.7 (24.8)	101.0 (24.9)
30	29.7 (8.5)	29.6 (8.5)	110	112.3 (25.9)	110.7 (26.6)
40	39.5 (10.6)	39.1 (10.4)	120	123.2 (27.5)	121.6 (28.8)
50	49.8 (12.6)	49.2 (12.5)	130	132.5 (28.1)	131.2 (29.7)
60	60.0 (15.0)	59.0 (14.7)	140	141.0 (28.9)	140.4 (32.0)
80	81.3 (20.2)	79.9 (20.0)	150	149.6 (30.0)	150.1 (34.1)

n=100 and S=52. The total $10^{4}$ simulated data sets are conditional on ρ and S as used in Figure 1. SD of $\hat{\rho }$ given in brackets.

**Figure 4:**
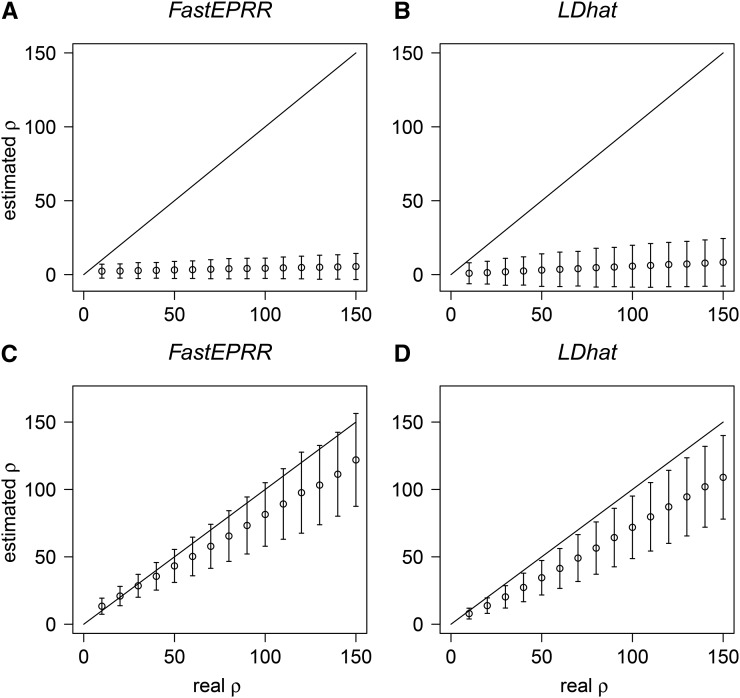
Comparisons of $\rho_{\text{FastEPRR}}$ (A) and (C), and $\rho_{\text{LDhat}}$ (B) and (D) under population bottleneck (A) and (B) and population exponential growth (C) and (D) conditions. n=100, S=52 and the time is scaled so that one unit represents $4N_{0}$ generations. For population bottleneck, we assume that duration $t_{1}=0.01$, and that the time of bottleneck ended $t_{0}=0.001$, and $N_{0}/N_{1}=100$, where $N_{0}$ is the effective population size before, and after, the bottleneck, and $N_{1}$ is the effective population size during the bottleneck. For population exponential growth, expansion time t=0.1, and $N_{0}/N_{1}=5$, where $N_{0}$ and $N_{1}$ are the current and ancestral effective population sizes, respectively.

**Figure 5:**
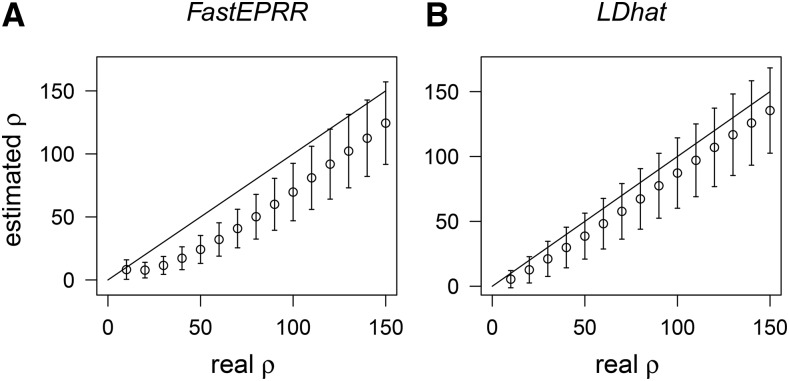
Comparison of $\rho_{\text{FastEPRR}}$ (A) and $\rho_{\text{LDhat}}$ (B) under the hitchhiking model. n=100, S=52, 2Ns=200, and the time after the beneficial allele gets to fixation *τ=0.01* (in units of 4N generations), where N is the effective population size, s the selection coefficient.

As neutral demographic scenarios (Johnston and Cutler 2012; Kamm *et al.* 2015) and positive selection (Reed and Tishkoff 2006) may cause the false recognition of recombination hotspots, we investigated the performance of FastEPRR in such cases. For example, when a population bottleneck occurs, both genetic variation and population size are substantially reduced; thus, estimated ρ is reduced (Figure 4, A and B, and Figure S4) compared to the current population recombination rate ($4N_{0}r$, where $N_{0}$ is the current effective population size), and the variance of $\rho_{\text{FastEPRR}}$ is generally smaller than $\rho_{\text{LDhat}}$ (Figure 4, A and B). Similarly, estimated ρ is also reduced in exponential population growth scenarios compared to a current population recombination rate (Figure 4, C and D). Importantly, its variance remains similar with that calculated under the standard neutral model (Figure 1E); therefore, recombination hotspots revealed by FastEPRR might not be due to the confounding effect of demography. Indeed, as FastEPRR is based on coalescent simulations, it would be possible to infer $4N_{0}r$ when demography parameters are estimated (Gutenkunst *et al.* 2009; Li and Stephan 2006; Li and Durbin 2011), but this is beyond the scope of this study. Positive selection reduces the DNA polymorphism level at linked neutral loci via the hitchhiking effect so a reduced ρ is expected (Figure 5) when compared to a population recombination rate estimated with the standard neutral model. Thus, positive selection cannot explain the recombination hotspots revealed by FastEPRR.

**Figure 6:**
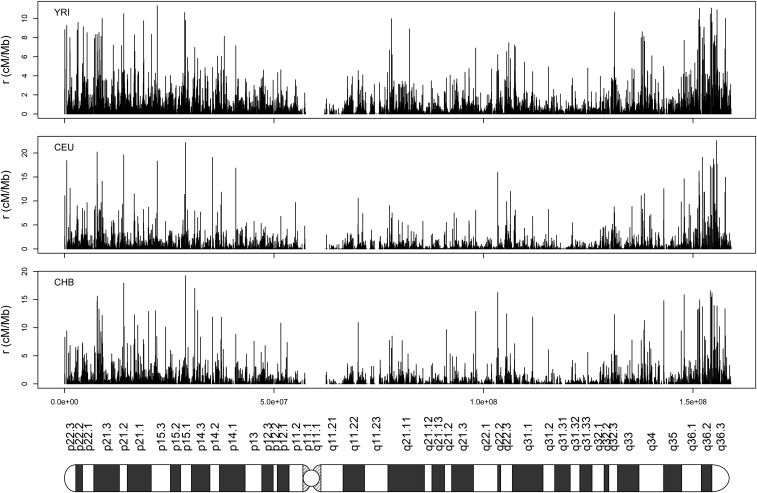
Recombination rates of chromosome 7 for three human populations of African (YRI), European (CEU), and East Asian (CHB) ancestry at a 50-kb scale. The cartoon at the bottom is a visualization of the chromosome.

**Figure 7:**
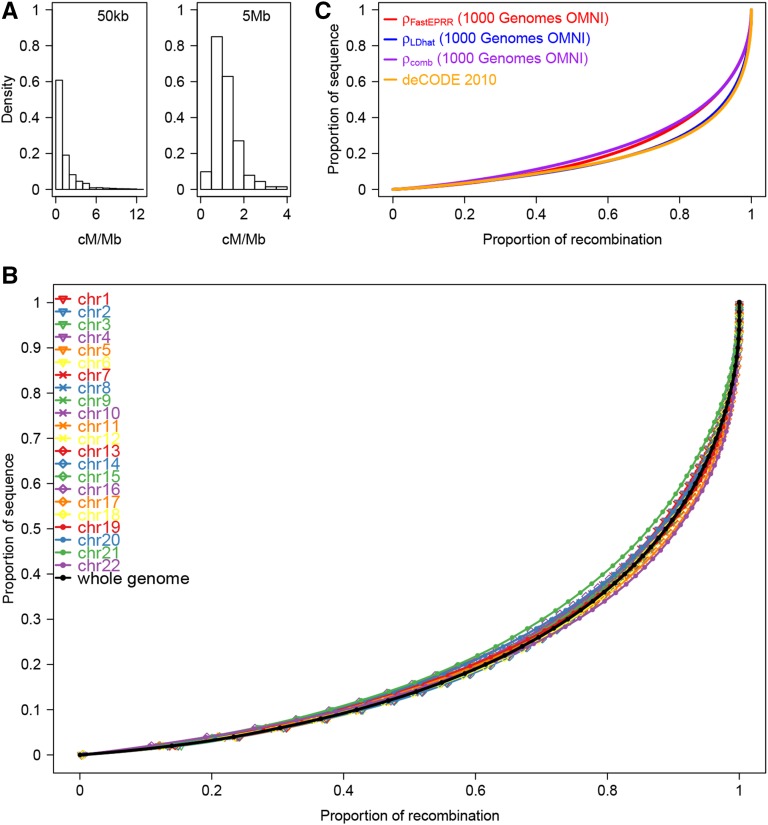
Recombination rate in the African population (YRI). (A) Histograms of the recombination rate for the whole autosomal genome at 50-kb and 5-Mb scales, respectively. (B) Proportion of recombination in different fractions of the sequence. Each colored line represents one chromosome, while the black line denotes the whole autosomal genome. (C) Concentration of recombination in a small proportion for the four genetic maps.

Our analysis of the 1000 Genomes OMNI data set (Altshuler *et al.* 2012) to estimate genome-wide $\rho_{\text{FastEPRR}}$ (chr1-chr22) in three human populations (*i.e.*, African, YRI, European, CEU, and East Asian, CHB, ancestry, see above) shows that the average ${\bar{\rho }}_{\text{FastEPRR}}(=4N_{e}\bar{r})$ per megabase in each case is 939.66 (YRI), 474.31 (CEU), and 544.75 (CHB). Using the 2010 deCODE family-based genetic map, average $\bar{r}$ for each population is thus 1.1703 cM/Mb (YRI), 1.1702 cM/Mb (CEU), and 1.1704 cM/Mb (CHB). Using estimates for $N_{e}$ of 20,073 (YRI), 10,133 (CEU) and 11,636 (CHB), the population recombination rate $\rho_{\text{FastEPRR}}$ can be converted as the recombination rate $r_{\text{FastEPRR}}$. As an example, we show recombination rates ($r_{\text{FastEPRR}}$) for chromosome 7 at the 50-kb scale for the YRI, CEU, and CHB populations (Figure 6), while recombination rates for the 22 autosomes are given in Figure S5. Recombination rates show a large degree of along-chromosome variation in the YRI population (Figure 7A), an overall trend that persists in all three populations (Figure 6). In the YRI population, the vast majority of recombination events occur in a small fraction of the sequence, *i.e.*, 70% of recombination events occur in 30% of the sequence (Figure 7B). On the other hand, recombination activity in the CEU and CHB populations is more concentrated (Figure S6), in agreement with previous findings (Altshuler *et al.* 2010).

We compared the $\rho_{\text{FastEPRR}}$ map with the one from $\rho_{\text{LDhat}}$ based on both the same data (Altshuler *et al.* 2012), and the 2010 deCODE family-based map (Kong *et al.* 2010). Overall, the $\rho_{\text{FastEPRR}}$ map is slightly more concentrated than the other two (Figure 7C and Figure S6), which means that $\rho_{\text{FastEPRR}}$ is more conservative in detecting recombination hotspots. We also calculated pairwise Pearson correlation coefficients for the three genetic maps of the three populations at 50-kb and 5-Mb scales (Table 3 and Figure S7); comparing the $\rho_{\text{FastEPRR}}$ and $\rho_{\text{LDhat}}$ maps, these coefficients range between 0.729 and 0.903 at the 50-kb scale, and between 0.929 and 0.987 at the 5-Mb scale. Thus, the two maps are highly correlated with one another, and also have a similar correlation coefficient to the 2010 deCODE map. Indeed, the correlation between estimates using FastEPRR and LDhat could be improved further if we consider the effect of variable recombination rates within windows using FastEPRR. Taking this into account, the Pearson correlation coefficients of the $\rho_{\text{FastEPRR}}$ and $\rho_{\text{LDhat}}$ maps for the YRI, CEU, and CHB populations at a 50-kb scale are 0.909, 0.813, and 0.770, respectively.

**Table 3:** Pairwise Pearson correlation coefficients among three genetic maps for three human populations

	FastEPRR.YRI	FastEPRR.CEU	FastEPRR.CHB	LDhat.YRI	LDhat.CEU	LDhat.CHB	deCODE
FastEPRR.YRI	1	0.969	0.955	0.987	0.964	0.964	0.859
FastEPRR.CEU	0.808	1	0.965	0.957	0.956	0.951	0.847
FastEPRR.CHB	0.793	0.845	1	0.939	0.929	0.939	0.840
LDhat.YRI	0.903	0.774	0.751	1	0.974	0.974	0.870
LDhat.CEU	0.791	0.803	0.729	0.826	1	0.974	0.866
LDhat.CHB	0.794	0.762	0.754	0.830	0.852	1	0.876
deCODE	0.626	0.601	0.554	0.641	0.679	0.655	1

Pairwise Pearson correlation coefficients among three genetic maps for three populations (YRI, CEU, and CHB) at a 50-kb scale are shown in the lower left triangle, while those at a 5-Mb scale are in the upper right triangle. FastEPRR.YRI, FastEPRR.CEU and FastEPRR.CHB denote the $\rho_{\text{FastEPRR}}$ maps for the three populations, LDhat.YRI, LDhat.CEU, and LDhat.CHB denote the $\rho_{\text{LDhat}}$ maps, and deCODE denotes the 2010 deCODE family-based map.

We also established another genetic map using $\rho_{\text{comb}}$ (by averaging $\rho_{\text{FastEPRR}}$ and $\rho_{\text{LDhat}}$) (Figure 7C and Figure S6) as this provides the most accurate estimate for recombination rate. In this case, the Pearson correlation coefficients between the $\rho_{\text{comb}}$ map and the 2010 deCODE map are 0.867 (YRI), 0.865 (CEU), and 0.869 (CHB) at a 5-Mb scale.

Complete genome-wide analysis of each population took less than 3 d on a single computer with a normal AMD Opteron(tm) 800 MHz processor using a single core (Table S3). Computing time for the genome-wide analysis of the YRI, CEU, and CHB populations (using a sliding window length of 50 kb) was 66.3, 45.7, and 49.0 hr, respectively. Indeed, if a small computer cluster (*i.e.*, 12 CPUs with four cores per CPU) was used, each analysis could be completed within less than 4 hr, and this time could be further decreased if the number of nodes were increased. FastEPRR will thus prove a very useful piece of software for the analysis of genome wide polymorphism data from large samples, for example the UK10K project (Walter *et al.* 2015) and other projects.

## Discussion

In this study, we introduce FastEPRR, a very fast piece of software that estimates population recombination rates from intraspecific DNA polymorphism data. FastEPRR is a much improved extension of our previously proposed regression-based method (Lin *et al.* 2013) that can be supported by computer clusters and so is suitable for the analysis of population genomic data even when sample sizes are very large. Furthermore, the new software excludes the number of folded singletons (${\text{ξ}}_{1}^{\text{′}}$) because they have no effect on the number of different haplotypes as recombination rates increase (Figure S8). Our evaluation of the performance of FastEPRR with, and without, ${\text{ξ}}_{1}^{\text{′}}$ (Figure S9), shows almost the same results in terms of means and the SD of estimated recombination rate. In agreement with previous work (Hudson and Kaplan 1985), we show that ${\text{ξ}}_{1}^{\text{′}}$ provides little information about recombination.

We also demonstrate that FastEPRR is naturally robust to multiple hits, one very important feature as it has been argued that these cannot be underestimated when calculating recombination rate (McVean *et al.* 2002). Moreover, because FastEPRR is a coalescent-simulation-based approach, it can handle the missing data often encountered in genomic scale population data sets. Simulations show that the phasing process does not affect recombination rate estimates when ρ≤100. Indeed, when ρ>100, estimates are still unbiased, but their variance increases slightly as it may be difficult to infer haplotypes. As a result, a reasonable window size should be used when estimating $\rho_{\text{FastEPRR}}$.

Our simulations show that the FastEPRR software provides the same degree of accuracy as well-known composite-likelihood methods but requires very little computation time. Using a single CPU core, for example, FastEPRR took less than 3 d to analyze the 1000 Genomes OMNI data set (Altshuler *et al.* 2012), a task that would take LDhat years. The Pearson correlation coefficient between the $\rho_{\text{FastEPRR}}$ and $\rho_{\text{LDhat}}$ maps is between 0.929 and 0.987 at a 5-Mb scale.

We propose that $\rho_{\text{comb}}$ has the smallest variance, when compared with $\rho_{\text{FastEPRR}}$ and $\rho_{\text{LDhat}}$. Because $\rho_{\text{comb}}$ is the average of $\rho_{\text{FastEPRR}}$ and $\rho_{\text{LDhat}}$, computation time for $\rho_{\text{comb}}$ will be determined mainly by $\rho_{\text{LDhat}}$. Thus, $\rho_{\text{comb}}$ can be used when sample sizes are small, and it is not difficult to estimate $\rho_{\text{LDhat}}$ in such cases. However, we recommend using $\rho_{\text{FastEPRR}}$ at larger sample sizes.

Sample sizes are expected to increase dramatically as sequencing technologies advance and more and more organisms are investigated (Cao *et al.* 2011; Altshuler *et al.* 2012; Walter *et al.* 2015). Whole genomes, or exomes, of nearly 10,000 individuals are included in the UK10K project (Walter *et al.* 2015), and the rapid construction of genetic maps is increasingly important to biological research. Next, we plan to apply the FastEPRR software to the UK10K data to establish the genetic map of the 10,000 individuals, a computational analysis we expect to take less than 2 wk. When complete, we will provide this map free on our website (http://www.picb.ac.cn/evolgen/softwares/) to facilitate other studies and to promote FastEPRR as a useful, fast, and effective tool for creating genetic maps and studying recombination hotspots in the genomic era.

## Supplementary Material

Supplemental Material

## References

[bib1] Altshuler D. Durbin R. M. Abecasis G. R. Bentley D. R. Chakravarti A., 2010 A map of human genome variation from population-scale sequencing. Nature 467(7319): 1061–1073. 20981092 10.1038/nature09534 PMC3042601

[bib2] Altshuler D. M. Durbin R. M. Abecasis G. R. Bentley D. R. Chakravarti A., 2012 An integrated map of genetic variation from 1,092 human genomes. Nature 491(7422): 56–65. 23128226 10.1038/nature11632 PMC3498066

[bib3] Auton A. McVean G., 2007 Recombination rate estimation in the presence of hotspots. Genome Res. 17(8): 1219–1227. 17623807 10.1101/gr.6386707 PMC1933511

[bib4] Auton A. Rui Li Y. Kidd J. Oliveira K. Nadel J., 2013 Genetic recombination is targeted towards gene promoter regions in dogs. PLoS Genet. 9(12): e1003984. 24348265 10.1371/journal.pgen.1003984 PMC3861134

[bib5] Baudat F. Buard J. Grey C. Fledel-Alon A. Ober C., 2010 PRDM9 is a major determinant of meiotic recombination hotspots in humans and mice. Science 327(5967): 836–840. 20044539 10.1126/science.1183439 PMC4295902

[bib6] Cao J. Schneeberger K. Ossowski S. Gunther T. Bender S., 2011 Whole-genome sequencing of multiple *Arabidopsis thaliana* populations. Nat. Genet. 43(10): 956–963. 21874002 10.1038/ng.911

[bib7] Coop G. Przeworski M., 2007 An evolutionary view of human recombination. Nat. Rev. Genet. 8(1): 23–34. 17146469 10.1038/nrg1947

[bib8] Ewing G. Hermisson J., 2010 MSMS: a coalescent simulation program including recombination, demographic structure and selection at a single locus. Bioinformatics 26(16): 2064–2065. 20591904 10.1093/bioinformatics/btq322 PMC2916717

[bib9] Fearnhead P. Donnelly P., 2001 Estimating recombination rates from population genetic data. Genetics 159(3): 1299–1318. 11729171 10.1093/genetics/159.3.1299 PMC1461855

[bib10] Fearnhead P. Donnelly P., 2002 Approximate likelihood methods for estimating local recombination rates. J. R. Stat. Soc. Series B Stat. Methodol. 64: 657–680.

[bib11] Fu Y. X. Li W. H., 1993 Statistical tests of neutrality of mutations. Genetics 133(3): 693–709. 8454210 10.1093/genetics/133.3.693 PMC1205353

[bib12] Griffiths R. C. Marjoram P., 1996 Ancestral inference from samples of DNA sequences with recombination. J. Comput. Biol. 3(4): 479–502. 9018600 10.1089/cmb.1996.3.479

[bib13] Gutenkunst R. N. Hernandez R. D. Williamson S. H. Bustamante C. D., 2009 Inferring the joint demographic history of multiple populations from multidimensional SNP frequency data. PLoS Genet. 5(10): e1000695. 10.1371/journal.pgen.1000695 PMC2760211 19851460

[bib14] Hernandez R. D. Kelley J. L. Elyashiv E. Melton S. C. Auton A., 2011 Classic selective sweeps were rare in recent human evolution. Science 331(6019): 920–924. 21330547 10.1126/science.1198878 PMC3669691

[bib15] Hill W. G. Robertson A., 1968 Linkage disequilibrium in finite populations. Theor. Appl. Genet. 38(6): 226–231. 24442307 10.1007/BF01245622

[bib16] Hothorn, T., P. Buehlmann, T. Kneib, M. Schmid, and B. Hofner, 2015 mboost: model-based boosting. Available at: http://CRAN.R-project.org/package=mboost. Accessed: February 25, 2015.

[bib17] Hudson R. R., 1985 The sampling distribution of linkage disequilibrium under an infinite allele model without selection. Genetics 109(3): 611–631. 3979817 10.1093/genetics/109.3.611 PMC1216291

[bib18] Hudson R. R., 1987 Estimating the recombination parameter of a finite population-model without selection. Genet. Res. 50(3): 245–250. 3443297 10.1017/s0016672300023776

[bib19] Hudson R. R., 2001 Two-locus sampling distributions and their application. Genetics 159(4): 1805–1817. 11779816 10.1093/genetics/159.4.1805 PMC1461925

[bib20] Hudson R. R., 2002 Generating samples under a Wright-Fisher neutral model of genetic variation. Bioinformatics 18(2): 337–338. 11847089 10.1093/bioinformatics/18.2.337

[bib21] Hudson R. R. Kaplan N. L., 1985 Statistical properties of the number of recombination events in the history of a sample of DNA sequences. Genetics 111(1): 147–164. 4029609 10.1093/genetics/111.1.147 PMC1202594

[bib22] Johnston H. R. Cutler D. J., 2012 Population demographic history can cause the appearance of recombination hotspots. Am. J. Hum. Genet. 90(5): 774–783. 22560089 10.1016/j.ajhg.2012.03.011 PMC3376637

[bib23] Kamm, J.A., J.P. Spence, J. Chan, and Y.S. Song, 2015 An exact algorithm and efficient importance sampling for computing two-locus likelihoods under variable population size. arXiv:1510.06017. Available at: http://adsabs.harvard.edu/abs/2015arXiv151006017K. Accessed: October 20, 2015.

[bib24] Kong A. Masson G. Frigge M. L. Gylfason A. Zusmanovich P., 2008 Detection of sharing by descent, long-range phasing and haplotype imputation. Nat. Genet. 40(9): 1068–1075. 19165921 10.1038/ng.216 PMC4540081

[bib25] Kong A. Thorleifsson G. Gudbjartsson D. F. Masson G. Sigurdsson A., 2010 Fine-scale recombination rate differences between sexes, populations and individuals. Nature 467(7319): 1099–1103. 20981099 10.1038/nature09525

[bib26] Kuhner M. K. Yamato J. Felsenstein J., 2000 Maximum likelihood estimation of recombination rates from population data. Genetics 156(3): 1393–1401. 11063710 10.1093/genetics/156.3.1393 PMC1461317

[bib27] Li H. Durbin R., 2011 Inference of human population history from individual whole-genome sequences. Nature 475(7357): 493–496. 21753753 10.1038/nature10231 PMC3154645

[bib28] Li H. P. Stephan W., 2005 Maximum-likelihood methods for detecting recent positive selection and localizing the selected site in the genome. Genetics 171(1): 377–384. 15972464 10.1534/genetics.105.041368 PMC1456529

[bib29] Li H. P. Stephan W., 2006 Inferring the demographic history and rate of adaptive substitution in *Drosophila*. PLoS Genet. 2(10): 1580–1589. 10.1371/journal.pgen.0020166 PMC1599771 17040129

[bib30] Li N. Stephens M., 2003 Modeling linkage disequilibrium and identifying recombination hotspots using single-nucleotide polymorphism data. Genetics 165(4): 2213–2233. 14704198 10.1093/genetics/165.4.2213 PMC1462870

[bib31] Lin K. Futschik A. Li H., 2013 A fast estimate for the population recombination rate based on regression. Genetics 194(2): 473–484. 23589457 10.1534/genetics.113.150201 PMC3664856

[bib32] Lu S. J. Zong C. H. Fan W. Yang M. Y. Li J. S., 2012 Probing meiotic recombination and aneuploidy of single sperm cells by whole-genome sequencing. Science 338(6114): 1627–1630. 23258895 10.1126/science.1229112 PMC3590491

[bib33] McVean G. Awadalla P. Fearnhead P., 2002 A coalescent-based method for detecting and estimating recombination from gene sequences. Genetics 160(3): 1231–1241. 11901136 10.1093/genetics/160.3.1231 PMC1462015

[bib34] Myers S. R. Griffiths R. C., 2003 Bounds on the minimum number of recombination events in a sample history. Genetics 163(1): 375–394. 12586723 10.1093/genetics/163.1.375 PMC1462432

[bib35] Nielsen R., 2000 Estimation of population parameters and recombination rates from single nucleotide polymorphisms. Genetics 154(2): 931–942. 10655242 10.1093/genetics/154.2.931 PMC1460954

[bib36] Ohta T. Kimura M., 1971 Linkage disequilibrium between two segregating nucleotide sites under the steady flux of mutations in a finite population. Genetics 68(4): 571–580. 5120656 10.1093/genetics/68.4.571 PMC1212677

[bib37] Price A. L. Tandon A. Patterson N. Barnes K. C. Rafaels N., 2009 Sensitive detection of chromosomal segments of distinct ancestry in admixed populations. PLoS Genet. 5(6): e1000519. 19543370 10.1371/journal.pgen.1000519 PMC2689842

[bib38] Pugach I. Matveyev R. Wollstein A. Kayser M. Stoneking M., 2011 Dating the age of admixture via wavelet transform analysis of genome-wide data. Genome Biol. 12(2): R19. 21352535 10.1186/gb-2011-12-2-r19 PMC3188801

[bib39] Reed F. A. Tishkoff S. A., 2006 Positive selection can create false hotspots of recombination. Genetics 172(3): 2011–2014. 16387873 10.1534/genetics.105.052183 PMC1456281

[bib40] Sattath S. Elyashiv E. Kolodny O. Rinott Y. Sella G., 2011 Pervasive adaptive protein evolution apparent in diversity patterns around amino acid substitutions in *Drosophila simulans*. PLoS Genet. 7(2): e1001302. 21347283 10.1371/journal.pgen.1001302 PMC3037414

[bib41] Shannon C. E., 1948 A mathematical theory of communication. Bell Syst. Tech. J. 27(4): 623–656.

[bib42] Stephens M. Scheet P., 2005 Accounting for decay of linkage disequilibrium in haplotype inference and missing-data imputation. Am. J. Hum. Genet. 76(3): 449–462. 15700229 10.1086/428594 PMC1196397

[bib43] Stephens M. Smith N. J. Donnelly P., 2001 A new statistical method for haplotype reconstruction from population data. Am. J. Hum. Genet. 68(4): 978–989. 11254454 10.1086/319501 PMC1275651

[bib44] Wall J. D., 2000 A comparison of estimators of the population recombination rate. Mol. Biol. Evol. 17(1): 156–163. 10666715 10.1093/oxfordjournals.molbev.a026228

[bib45] Walter K. Min J. L. Huang J. Crooks L. Memari Y., 2015 The UK10K project identifies rare variants in health and disease. Nature 526(7571): 82. 26367797 10.1038/nature14962 PMC4773891

[bib46] Wang Y. Rannala B., 2008 Bayesian inference of fine-scale recombination rates using population genomic data. Philos. Trans. R. Soc. Lond. B Biol. Sci. 363(1512): 3921–3930. 18852101 10.1098/rstb.2008.0172 PMC2607416

[bib47] Wang Y. Rannala B., 2009 Population genomic inference of recombination rates and hotspots. Proc. Natl. Acad. Sci. USA 106(15): 6215–6219. 19342488 10.1073/pnas.0900418106 PMC2669376

[bib48] Weiss K. M. Clark A. G., 2002 Linkage disequilibrium and the mapping of complex human traits. Trends Genet. 18: 19–24. 11750696 10.1016/s0168-9525(01)02550-1

