# Supplementary material for: New Software for the Fast Estimation of Population Recombination Rates (FastEPRR) in the Genomic Era
Source: G3 (Bethesda). 2016 Mar 29;6(6):1563–71. doi: 10.1534/g3.116.028233 (PMC4889653; doi:10.1534/g3.116.028233)
Supplement: Supplemental Material [file supp_6_6_1563__index.html]

New Software for the Fast Estimation of Population Recombination Rates (FastEPRR) in the Genomic Era — Supplemental Material 

# New Software for the Fast Estimation of Population Recombination Rates (FastEPRR) in the Genomic Era

## Supplemental Material for Gao *et al.*, 2016

**Files in this Data Supplement:**

- File S1 - (.pdf, 152 KB)
- Figure S1 - (.pdf, 135 KB)
- Figure S2 - (.pdf, 137 KB)
- Figure S3 - (.pdf, 140 KB)
- Figure S4 - (.pdf, 139 KB)
- Supplemental Legends - File contains legends for Supplemental File, Figures, and Tables. (.pdf, 134 KB)
- Figure S5 - (.pdf, 840 KB)
- Figure S6 - (.pdf, 64 KB)
- Figure S7 - (.pdf, 409 KB)
- Figure S8 - (.pdf, 143 KB)
- Figure S9 - (.pdf, 154 KB)
- Table S1 - (.pdf, 133 KB)
- Table S2 - (.pdf, 152 KB)
- Table S3 - (.pdf, 12 KB)
