# Supplementary material for: New Software for the Fast Estimation of Population Recombination Rates (FastEPRR) in the Genomic Era
Source: G3 (Bethesda). 2016 Mar 29;6(6):1563–71. doi: 10.1534/g3.116.028233 (PMC4889653; doi:10.1534/g3.116.028233)
Supplement: Supplemental Material [file supp_g3.116.028233_FileS1.pdf]

File S1. The method to consider variable recombination rates within windows

Given $\hat{\rho}_1$, $\hat{\rho}_2$ and $\hat{\rho}_3$ (Figure S1), the following pseudocode describes the solving procedure for considering the variable recombination rates within windows.

If ($\hat{\rho}_2 \geq (\hat{\rho}_1 + \hat{\rho}_3)$) {

#According to the constraint condition (2)

$x_1 = 0, x_2 = \frac{(\hat{\rho}_2 - \hat{\rho}_1 - \hat{\rho}_3)}{3} + \hat{\rho}_1, x_3 = \frac{(\hat{\rho}_2 - \hat{\rho}_1 - \hat{\rho}_3)}{3} + \hat{\rho}_3, x_4 = 0.$

} else {

#According to the constraint condition (3)

Let $C_2 = \hat{\rho}_1$, $C_3 = \hat{\rho}_2 - \hat{\rho}_1$, $C_4 = \hat{\rho}_3 - \hat{\rho}_2 + \hat{\rho}_1$, then $x_2 = C_2 - x_1$, $x_3 = C_3 + x_1$, $x_4 = C_4 - x_1$. And

$$f_2 = x_1 x_2 x_3 x_4 = x_1(C_2 - x_1)(C_3 + x_1)(C_4 - x_1)$$

$$= x_1^4 + (C_3 - C_2 - C_4)x_1^3 + (C_2 C_4 - C_3 C_4 - C_3 C_2)x_1^2 + C_2 C_3 C_4 x_1$$

Let f_2' be the derivative of f_2, then

$$f_2' = 4x_1^3 + 3(C_3 - C_2 - C_4)x_1^2 + 2(C_2 C_4 - C_3 C_4 - C_3 C_2)x_1 + C_2 C_3 C_4$$

According to the constraint condition (1), let $L = \max\{0, -C_3\}$ and $U = \min\{C_2, C_4\}$. We define $B = \{L, U, \text{all real roots of } f_2'\}$, $\forall b \in B$, $b^* = maximize\ f_2(b)$. Let $x_1 = b^*$, then $x_2 = C_2 - b^*, x_3 = C_3 + b^*$ and $x_4 = C_4 - b^*$.

}
