# Supplementary material for: New Software for the Fast Estimation of Population Recombination Rates (FastEPRR) in the Genomic Era
Source: G3 (Bethesda). 2016 Mar 29;6(6):1563–71. doi: 10.1534/g3.116.028233 (PMC4889653; doi:10.1534/g3.116.028233)
Supplement: Supplemental Material [file supp_g3.116.028233_FigureS1.pdf]

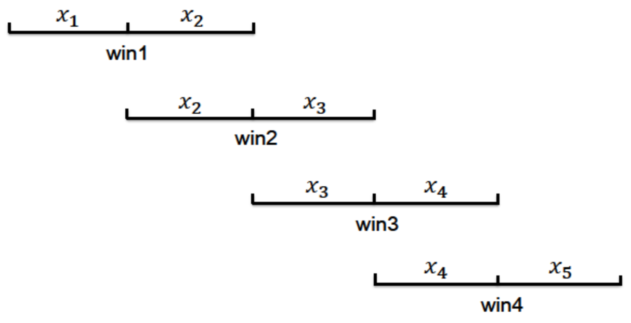

Figure S1 Illustration how to consider variable recombination rate within windows. Four overlapping sliding windows (*i.e.*, win1, win2, win3, win4) have the same window size, and the length of overlapping region for two continuous windows is half of its size. We have that the recombination rate of win1, win2, win3 and win4 is $x_1 + x_2$, $x_2 + x_3$, $x_3 + x_4$ and $x_4 + x_5$, respectively.
