# Supplementary material for: New Software for the Fast Estimation of Population Recombination Rates (FastEPRR) in the Genomic Era
Source: G3 (Bethesda). 2016 Mar 29;6(6):1563–71. doi: 10.1534/g3.116.028233 (PMC4889653; doi:10.1534/g3.116.028233)
Supplement: Supplemental Material [file supp_g3.116.028233_FigureS2.pdf]

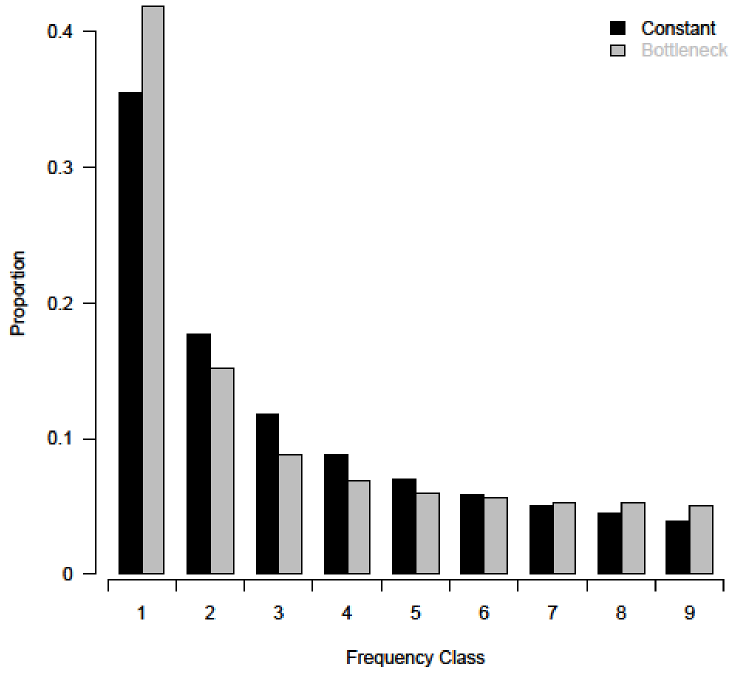

Figure S2 Mutation frequency spectrums (MFSs) under the constant population size model and the bottleneck scenario. $n = 10$, $\theta = 1{,}000$ and the time is scaled so that one unit represents $4N_0$ generations. For the bottleneck scenario, we assumed the duration of bottleneck $t_1 = 0.01$, the time of bottleneck ended $t_0 = 0.1$, and $N_0/N_1 = 10$, where N_0 is the effective population size before and after the bottleneck, N_1 the effective population size during the bottleneck. The MFS was estimated from 10^5 simulated data sets.
