# Supplementary material for: New Software for the Fast Estimation of Population Recombination Rates (FastEPRR) in the Genomic Era
Source: G3 (Bethesda). 2016 Mar 29;6(6):1563–71. doi: 10.1534/g3.116.028233 (PMC4889653; doi:10.1534/g3.116.028233)
Supplement: Supplemental Material [file supp_g3.116.028233_FigureS3.pdf]

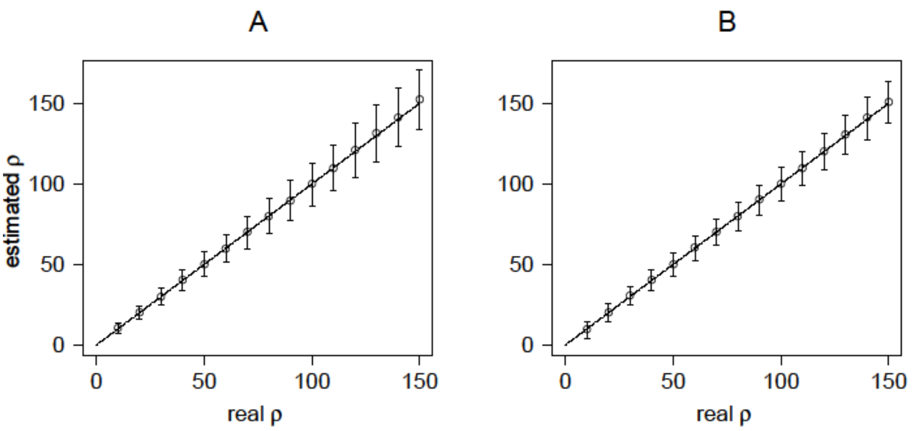

Figure S3 Comparisons of $\rho_{FastEPRR}$ when the sample size is very large ($n = 1000$). The mean and the standard deviation of $\hat{\rho}$ was estimated from 2,000 simulated data conditional on ρ and $S =$ 75 (A) or 375 (B).
