# Supplementary material for: New Software for the Fast Estimation of Population Recombination Rates (FastEPRR) in the Genomic Era
Source: G3 (Bethesda). 2016 Mar 29;6(6):1563–71. doi: 10.1534/g3.116.028233 (PMC4889653; doi:10.1534/g3.116.028233)
Supplement: Supplemental Material [file supp_g3.116.028233_FigureS4.pdf]

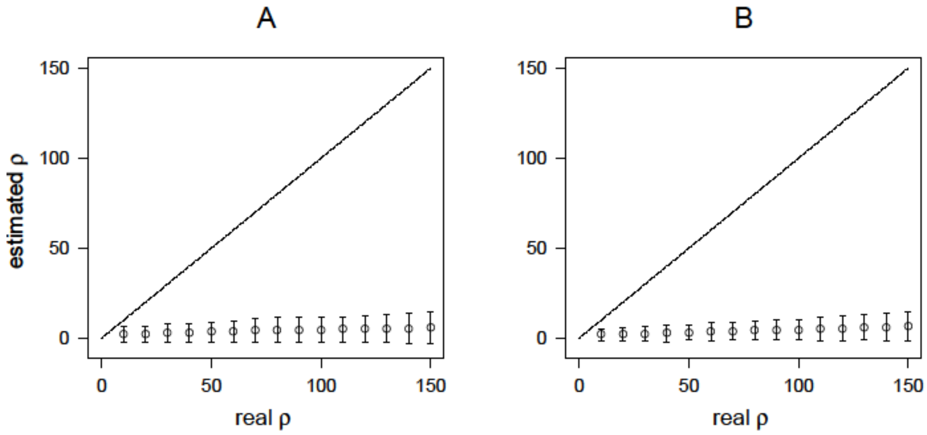

Figure S4 Comparing the performance of FastEPRR under the population bottleneck model with fixed $S = 52$ (A) and $\xi_2' = 5, \xi_x' = 37$ (B). We assumed $n = 100$, the duration of bottleneck $t_1 = 0.01$, the time of bottleneck ended $t_0 = 0.001$, and $N_0/N_1 = 100$, where N_0 is the effective population size before and after the bottleneck, N_1 the effective population size during the bottleneck, and the time is scaled so that one unit represents $4N_0$ generations.
