# Supplementary material for: New Software for the Fast Estimation of Population Recombination Rates (FastEPRR) in the Genomic Era
Source: G3 (Bethesda). 2016 Mar 29;6(6):1563–71. doi: 10.1534/g3.116.028233 (PMC4889653; doi:10.1534/g3.116.028233)
Supplement: Supplemental Material [file supp_g3.116.028233_SupplementalLegends.pdf]

File S1. The method to consider variable recombination rates within windows

Table S1 RMSE of $\rho_{FastEPRR}$ when the sample size increases. The RMSE of $\hat{\rho}$ was estimated from 2,000 simulated data conditional on real $\rho = 50$ and $S = 75$.

Table S2 Comparison of $\rho_{FastEPRR}$, ρ_{gam}, ρ_{LDhat} and ρ_{comb} for each sample size measured by RMSE. The parameters in Figure 1 were used, unless noted otherwise.

Table S3 Computing time to analyze the genome-wide polymorphism data for three human populations. The unit of measurement is given in hours (h). A single CPU core of a computer with an AMD Opteron(tm) 800MHz processor was used. As expected, the waiting/computing time will be dramatically shortened when a computer cluster is available.

Figure S1. Illustration how to consider variable recombination rate within windows. Four overlapping sliding windows (*i.e.*, win1, win2, win3, win4) have the same window size, and the length of overlapping region for two continuous windows is half of its size. We have that the recombination rate of win1, win2, win3 and win4 is $x_1 + x_2$, $x_2 + x_3$, $x_3 + x_4$ and $x_4 + x_5$, respectively.

Figure S2. Mutation frequency spectrums (MFSs) under the constant population size model and the bottleneck scenario. $n = 10$, $\theta = 1{,}000$ and the time is scaled so that one unit represents $4N_0$ generations. For the bottleneck scenario, we assumed the duration of bottleneck $t_1 = 0.01$, the time of bottleneck ended $t_0 = 0.1$, and $N_0/N_1 = 10$, where N_0 is the effective population size before and after the bottleneck, N_1 the effective population size during the bottleneck. The MFS was estimated from 10^5 simulated data sets.

Figure S3. Comparisons of $\rho_{FastEPRR}$ when the sample size is very large ($n = 1000$). The mean and the standard deviation of $\hat{\rho}$ was estimated from 2,000 simulated data conditional on ρ and $S =$ 75 (A) or 375 (B).

Figure S4. Comparing the performance of FastEPRR under the population bottleneck model with fixed $S = 52$ (A) and $\xi_2' = 5, \xi_x' = 37$ (B). We assumed $n = 100$, the duration of bottleneck $t_1 = 0.01$, the time of bottleneck ended $t_0 = 0.001$, and $N_0/N_1 = 100$, where N_0 is the effective population size before and after the bottleneck, N_1 the effective population size during the bottleneck, and the time is scaled so that one unit represents $4N_0$ generations.

Figure S5. Recombination rates of the 22 autosomes for three human populations of African (YRI), European (CEU) and East Asian (CHB) ancestry at a 50-kb scale. The cartoon at the bottom is a visualization of the chromosome.

Figure S6. Recombination rate in European (CEU) (A, B, C) and East Asian (CHB) (D, E, F). Histograms of the recombination rate for whole autosomal genome at 50-kb scale and 5-Mb scales (A, D). Proportion of recombination in different fractions of sequence. Each colored line represents one chromosome and the black line denotes the whole autosomal genome (B, E). Concentration of recombination in a small proportion for the four genetic maps (C, F).

Figure S7. Comparisons of $\rho_{FastEPRR}$ and ρ_{LDhat} maps at 50-kb and 5-Mb scales for African (YRI) (A, B), European (CEU) (C, D) and East Asian (CHB) (E, F), respectively. The scatter plots show the comparison of the whole genome and 22 autosomes.

Figure S8. The relationship between H and ρ under the condition of ξ_1', ξ_2', ξ_x' and S separately. $n = 100, S = 52, \xi_1' = 52, \xi_2' = 52, \xi_x' = 52$. The open circles represent the mean of

all H for each $\rho \in [10, 400]$.

Figure S9. Comparisons of $\boldsymbol{\rho_{FastEPRR}}$ with and without $\boldsymbol{\xi_1'}$. $\boldsymbol{\hat{\rho}}$ was estimated without $\boldsymbol{\xi_1'}$ (A-C) and with $\boldsymbol{\xi_1'}$ (D-F) for the sample sizes $\boldsymbol{n = 50}$ (A, D), $\boldsymbol{n = 100}$ (B, E) and $\boldsymbol{n = 200}$ (C, F). The number of segregating site $S = 45$ $(n = 50)$, 52 $(n = 100)$ and 59 $(n = 200)$.
