# Supplementary material for: New Software for the Fast Estimation of Population Recombination Rates (FastEPRR) in the Genomic Era
Source: G3 (Bethesda). 2016 Mar 29;6(6):1563–71. doi: 10.1534/g3.116.028233 (PMC4889653; doi:10.1534/g3.116.028233)
Supplement: Supplemental Material [file supp_g3.116.028233_FigureS5.pdf]

chr1

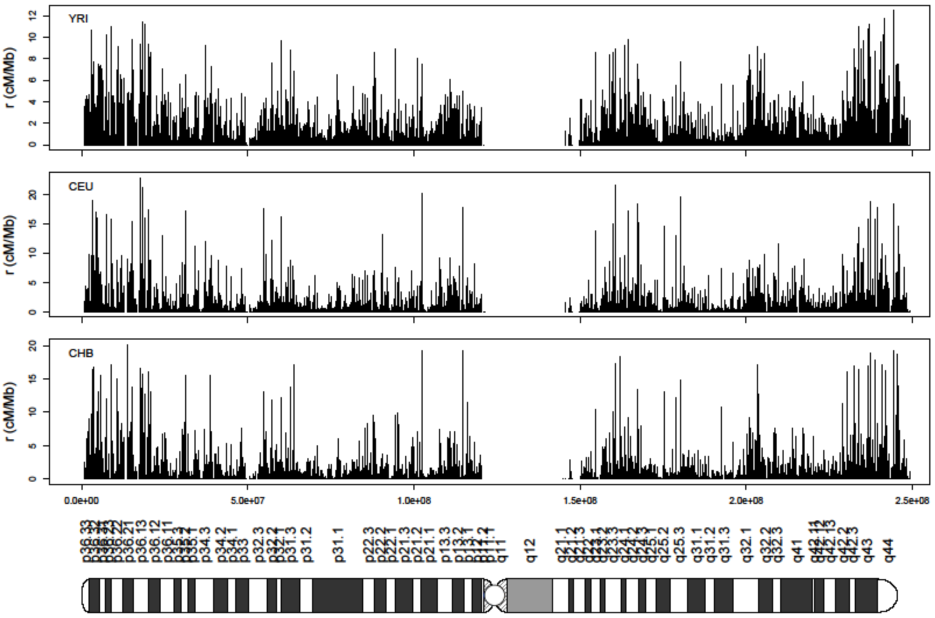
YRI
CEU
CHB
r (cM/Mb)
0.0e+00
5.0e+07
1.0e+08
1.5e+08
2.0e+08
2.5e+08

chr2

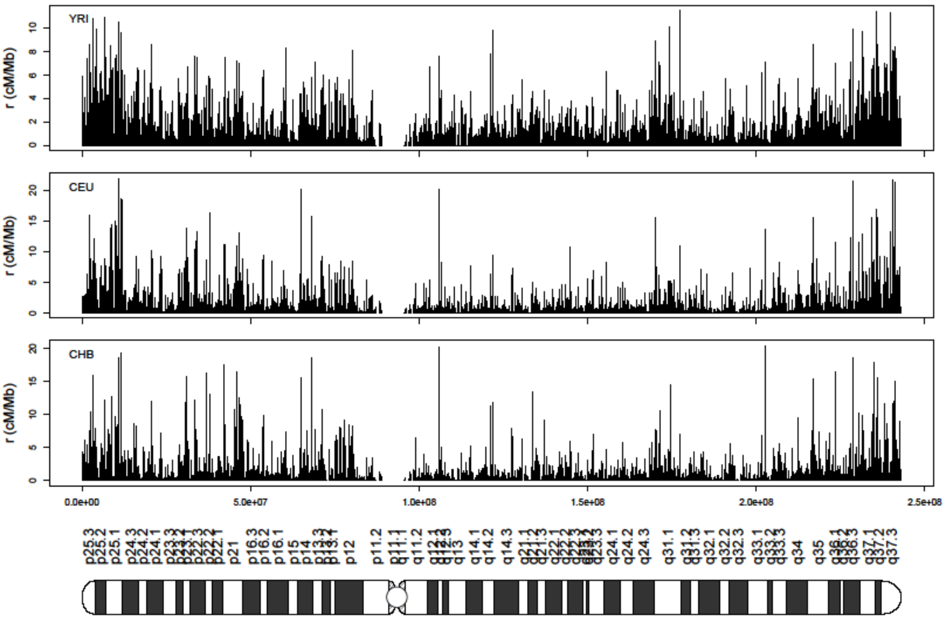
YRI
CEU
CHB
r (cM/Mb)
0.0e+00
5.0e+07
1.0e+08
1.5e+08
2.0e+08
2.5e+08

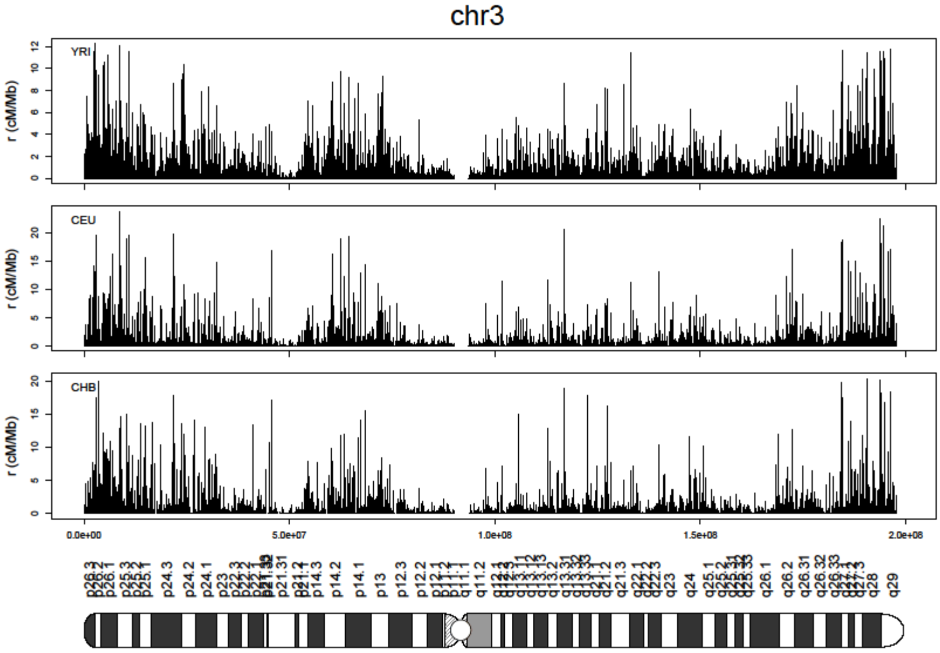
chr3
YRI
CEU
CHB
r (cM/Mb)
0.0e+00
5.0e+07
1.0e+08
1.5e+08
2.0e+08
p26.3
q29

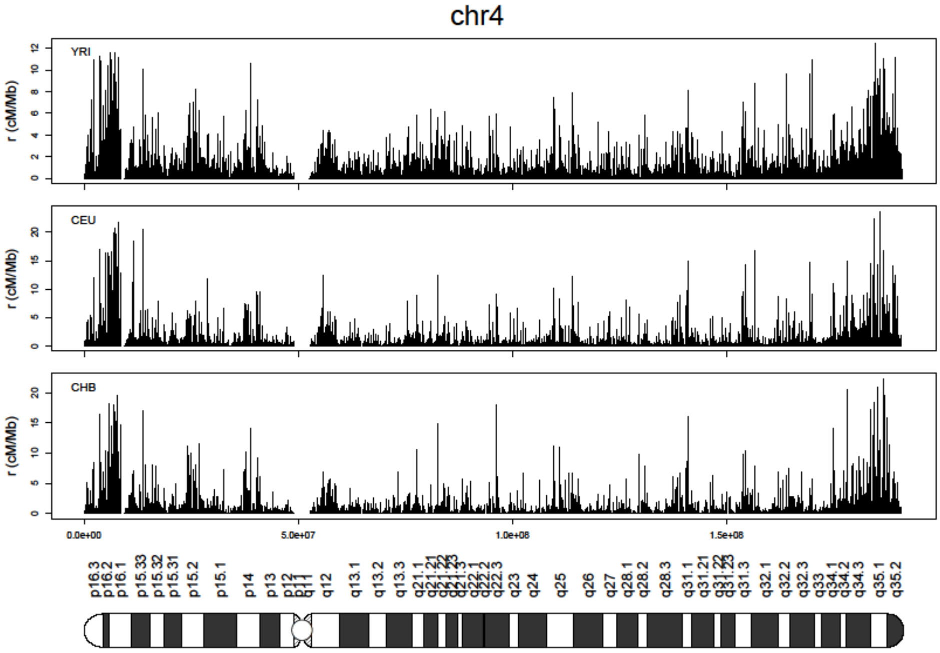
chr4
YRI
CEU
CHB
r (cM/Mb)
0.0e+00
5.0e+07
1.0e+08
1.5e+08
p16.3
q35.2

chr5

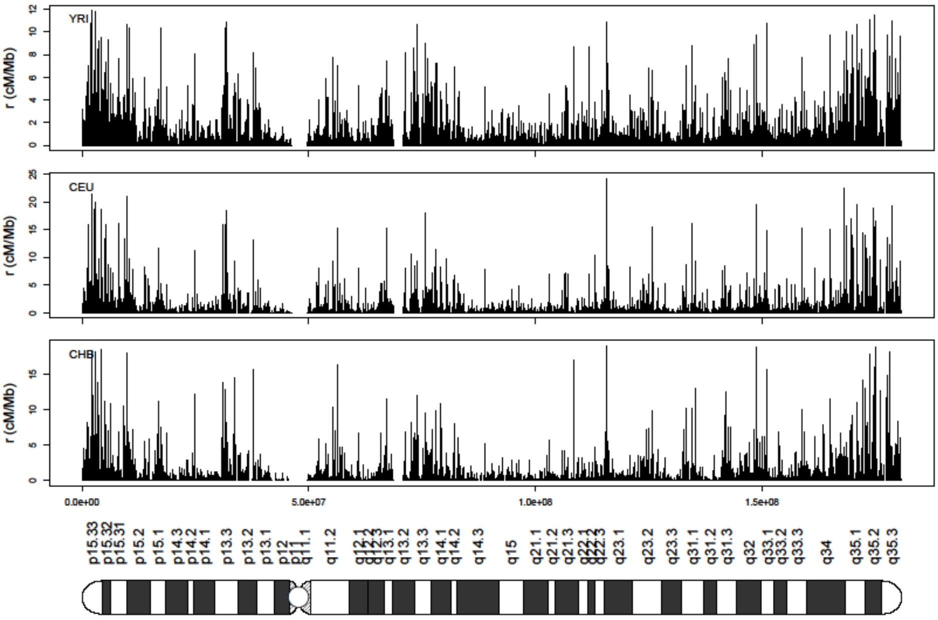
YRI
CEU
CHB
r (cM/Mb)
0.0e+00
5.0e+07
1.0e+08
1.5e+08

chr6

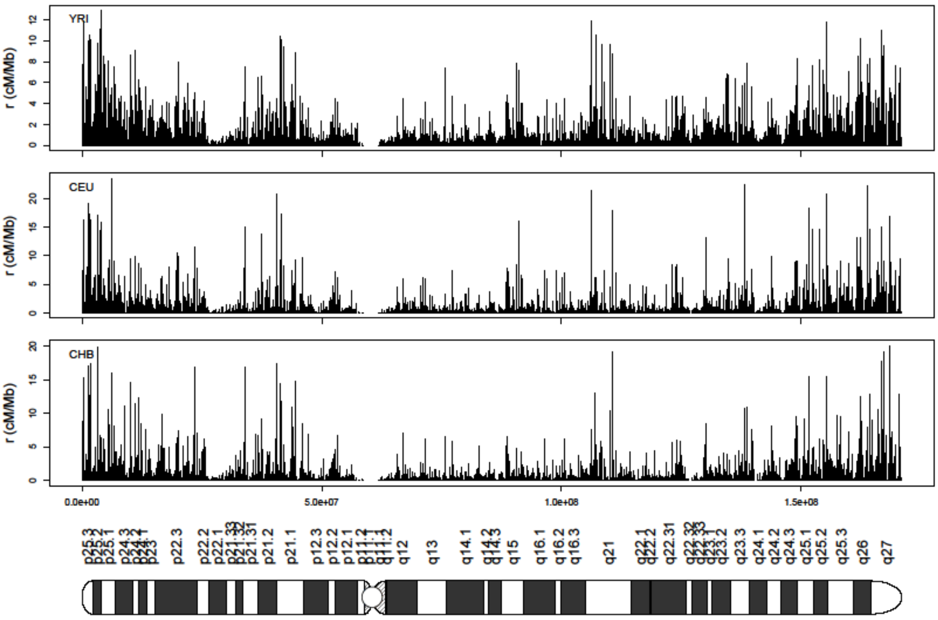
YRI
CEU
CHB
r (cM/Mb)
0.0e+00
5.0e+07
1.0e+08
1.5e+08

chr7

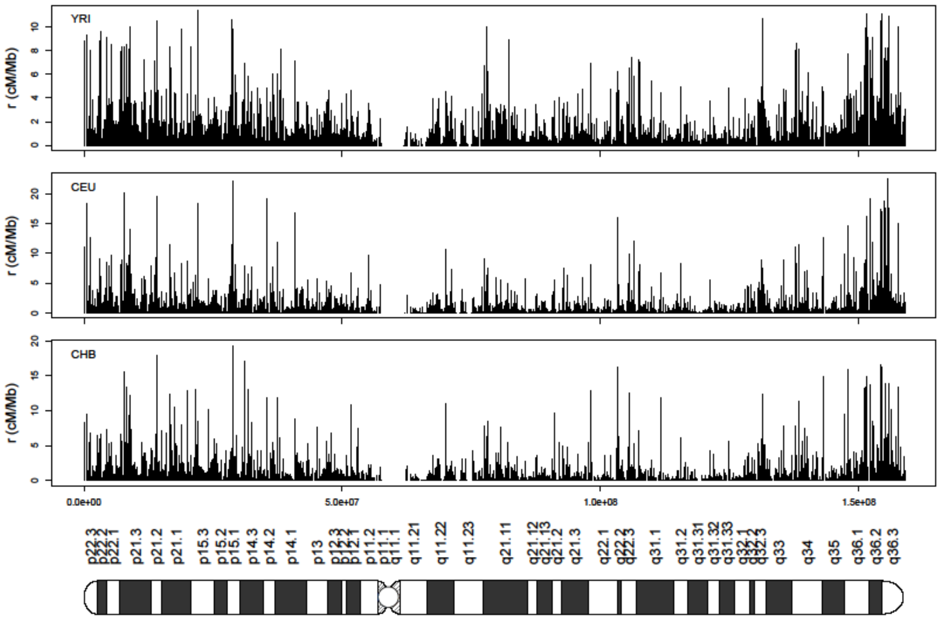
YRI
CEU
CHB
r (cM/Mb)
0.0e+00
5.0e+07
1.0e+08
1.5e+08

chr8

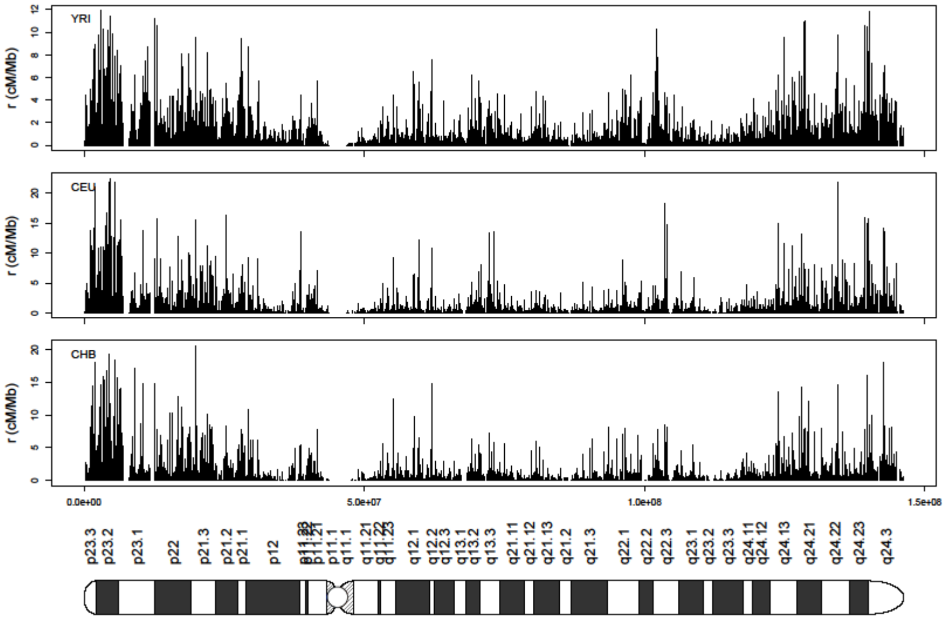
YRI
CEU
CHB
r (cM/Mb)
0.0e+00
5.0e+07
1.0e+08
1.5e+08

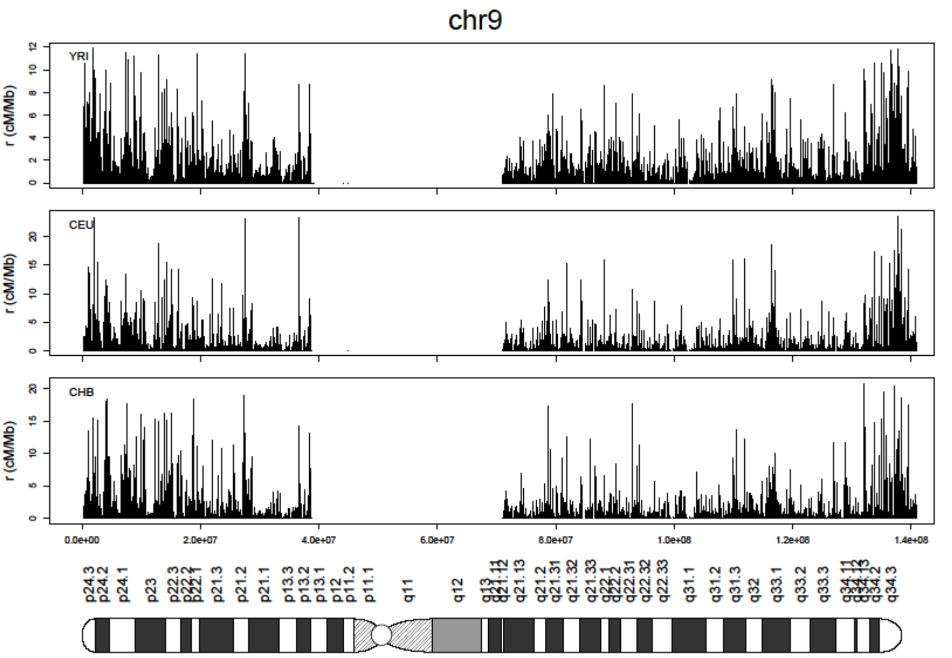

chr9
YRI
CEU
CHB
r (cM/Mb)
0.0e+00
2.0e+07
4.0e+07
6.0e+07
8.0e+07
1.0e+08
1.2e+08
1.4e+08

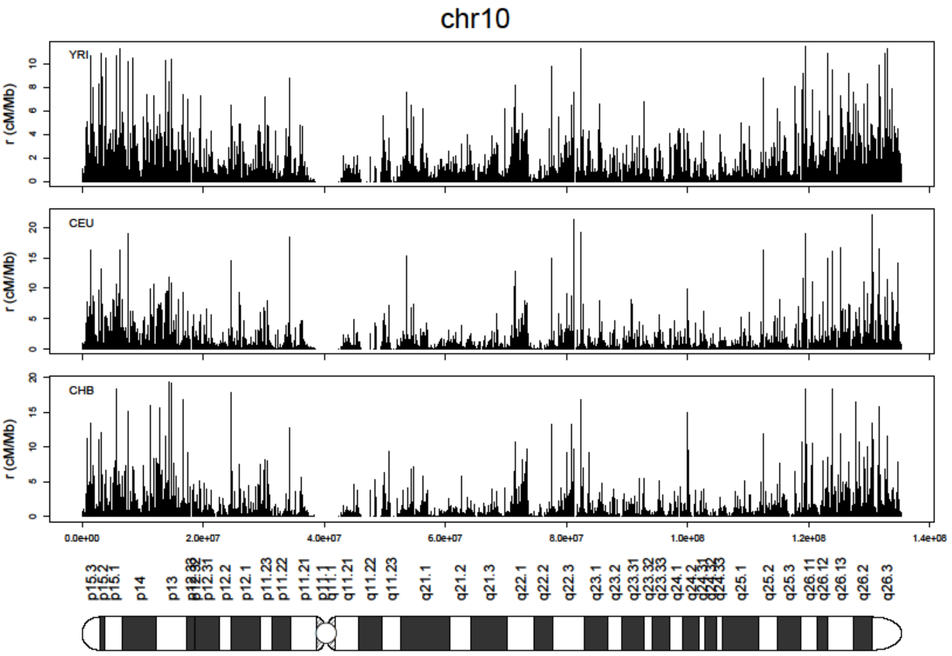

chr10
YRI
CEU
CHB
r (cM/Mb)
0.0e+00
2.0e+07
4.0e+07
6.0e+07
8.0e+07
1.0e+08
1.2e+08
1.4e+08

chr11

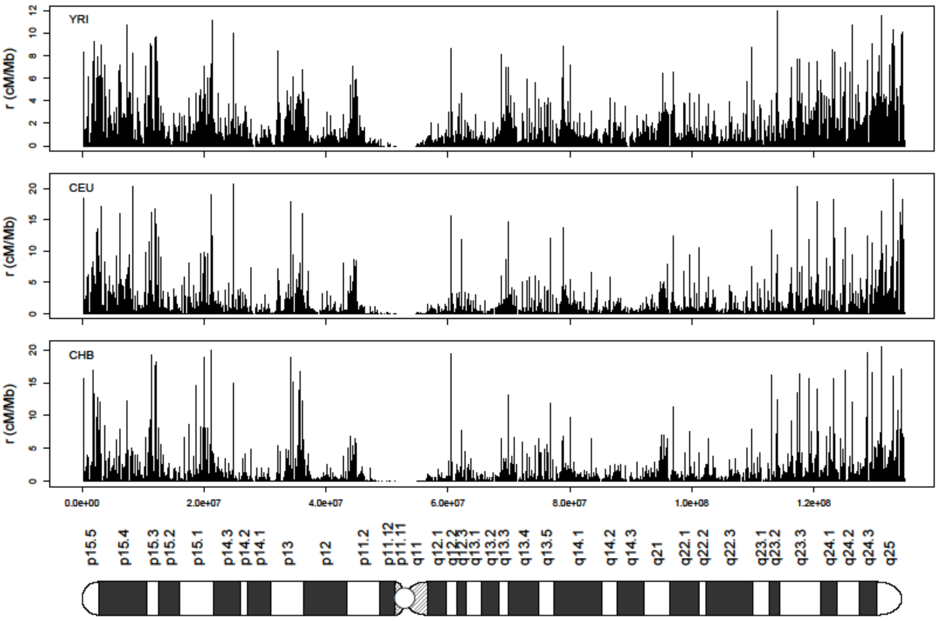
YRI
CEU
CHB
r (cM/Mb)
0.0e+00
2.0e+07
4.0e+07
6.0e+07
8.0e+07
1.0e+08
1.2e+08
p15.5
p15.4
p15.3
p15.2
p15.1
p14.3
p14.2
p14.1
p13
p12
p11.2
p11.12
p11.11
q11
q12.1
q13.1
q13.2
q13.3
q13.4
q13.5
q14.1
q14.2
q14.3
q21
q22.1
q22.2
q22.3
q23.1
q23.2
q23.3
q24.1
q24.2
q24.3
q25

chr12

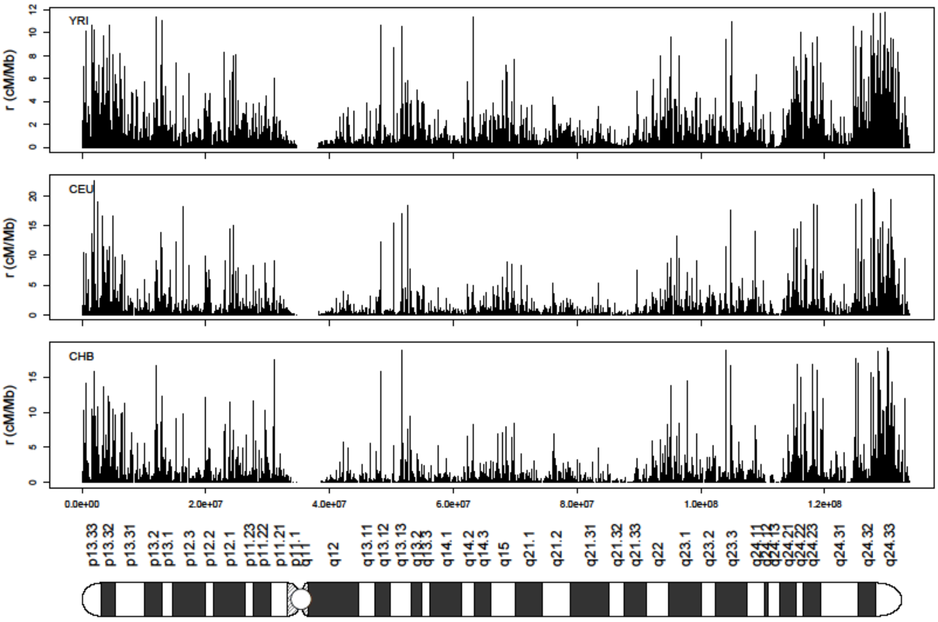
YRI
CEU
CHB
r (cM/Mb)
0.0e+00
2.0e+07
4.0e+07
6.0e+07
8.0e+07
1.0e+08
1.2e+08
p13.33
p13.32
p13.31
p13.2
p13.1
p12.3
p12.2
p12.1
p11.23
p11.22
p11.21
q12
q13.11
q13.12
q13.13
q14.1
q14.2
q14.3
q15
q21.1
q21.2
q21.31
q21.32
q21.33
q22
q23.1
q23.2
q23.3
q24.31
q24.32
q24.33

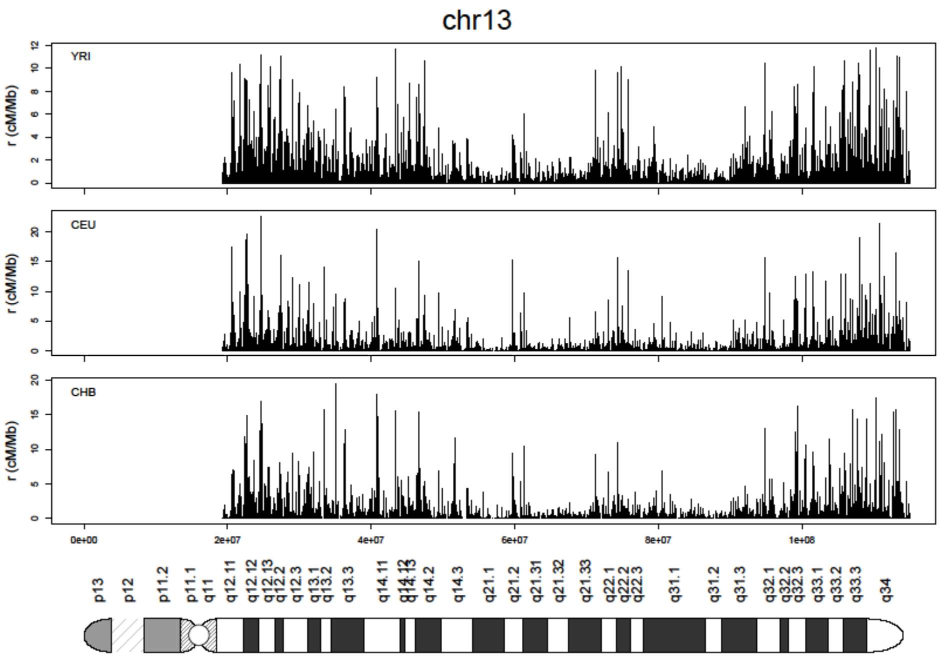
chr13
YRI
CEU
CHB
r (cM/Mb)
0e+00
2e+07
4e+07
6e+07
8e+07
1e+08

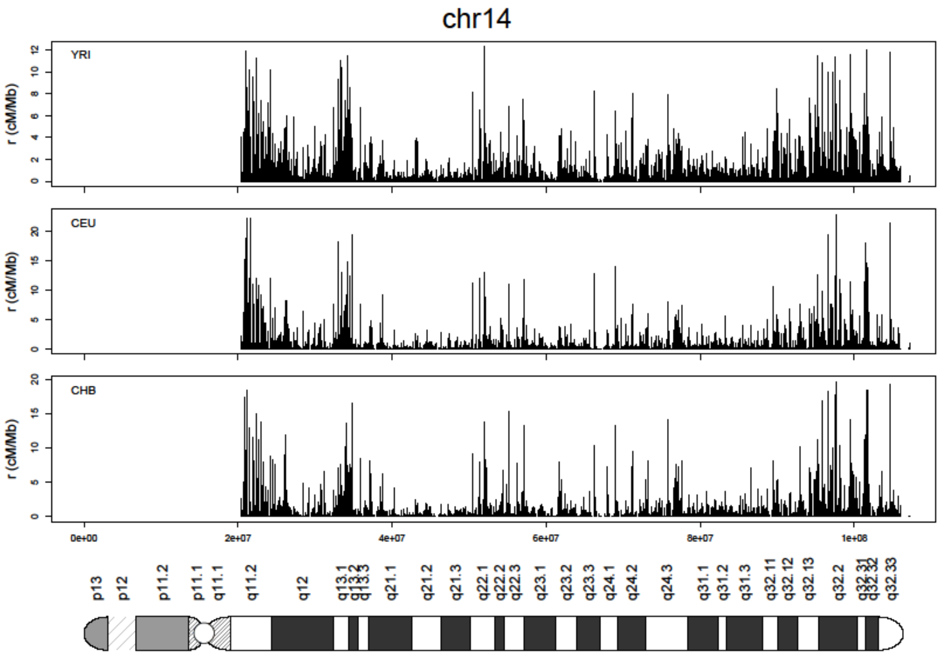
chr14
YRI
CEU
CHB
r (cM/Mb)
0e+00
2e+07
4e+07
6e+07
8e+07
1e+08

chr15

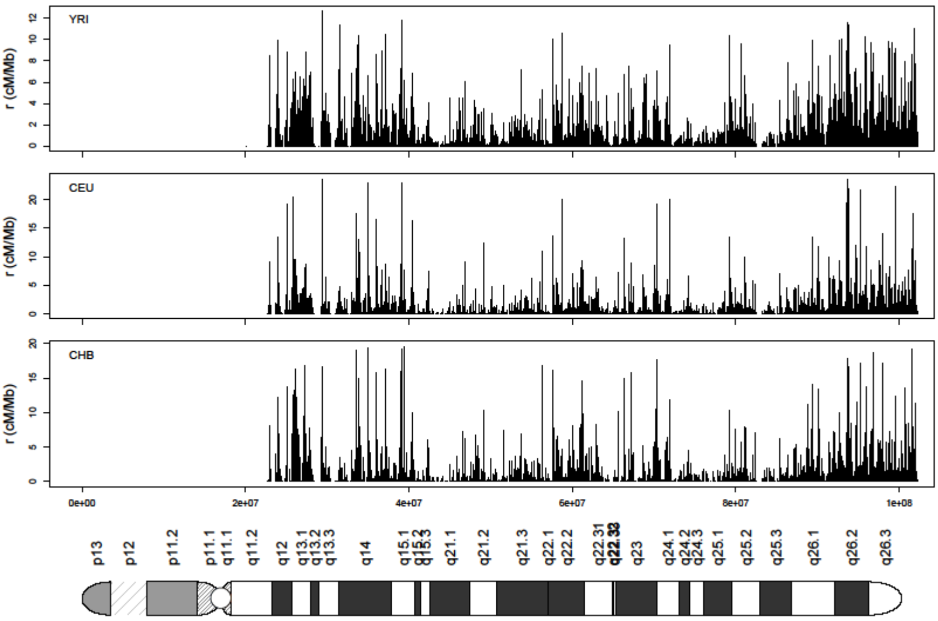
YRI
CEU
CHB
r (cM/Mb)
0e+00
2e+07
4e+07
6e+07
8e+07
1e+08
p13
p12
p11.2
p11.1
q11.1
q11.2
q12
q13.1
q13.2
q13.3
q14
q15.1
q15.2
q15.3
q21.1
q21.2
q21.3
q22.1
q22.2
q22.31
q23
q24.1
q24.2
q24.3
q25.1
q25.2
q25.3
q26.1
q26.2
q26.3

chr16

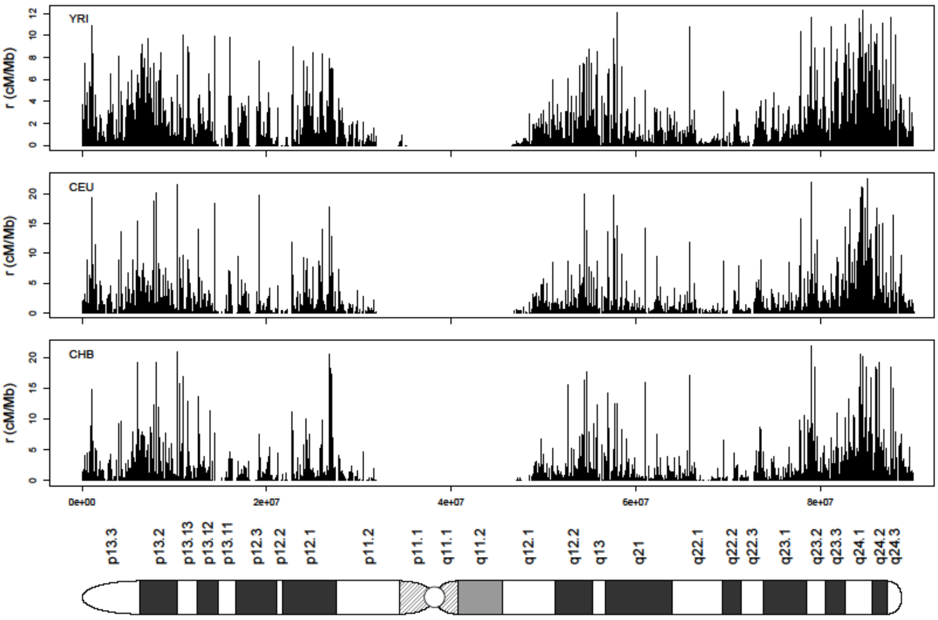
YRI
CEU
CHB
r (cM/Mb)
0e+00
2e+07
4e+07
6e+07
8e+07
p13.3
p13.2
p13.13
p13.12
p13.11
p12.3
p12.2
p12.1
p11.2
p11.1
q11.1
q11.2
q12.1
q12.2
q13
q21
q22.1
q22.2
q22.3
q23.1
q23.2
q23.3
q24.1
q24.2
q24.3

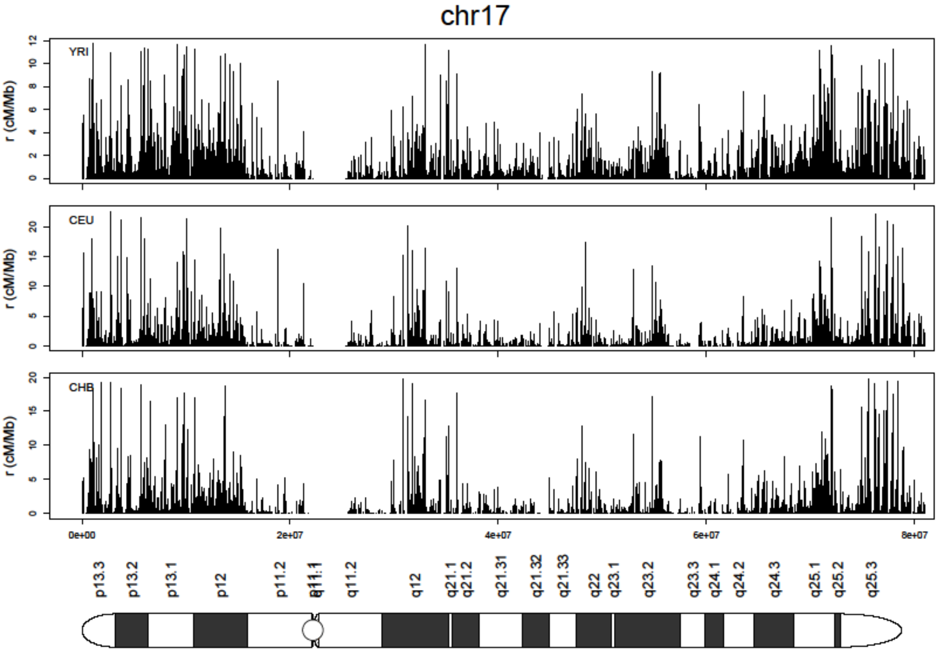
chr17
YRI
CEU
CHB
r (cM/Mb)
0e+00
2e+07
4e+07
6e+07
8e+07
p13.3
p13.2
p13.1
p12
p11.2
p11.1
q11.2
q12
q21.1
q21.2
q21.31
q21.32
q21.33
q22
q23.1
q23.2
q23.3
q24.1
q24.2
q24.3
q25.1
q25.2
q25.3

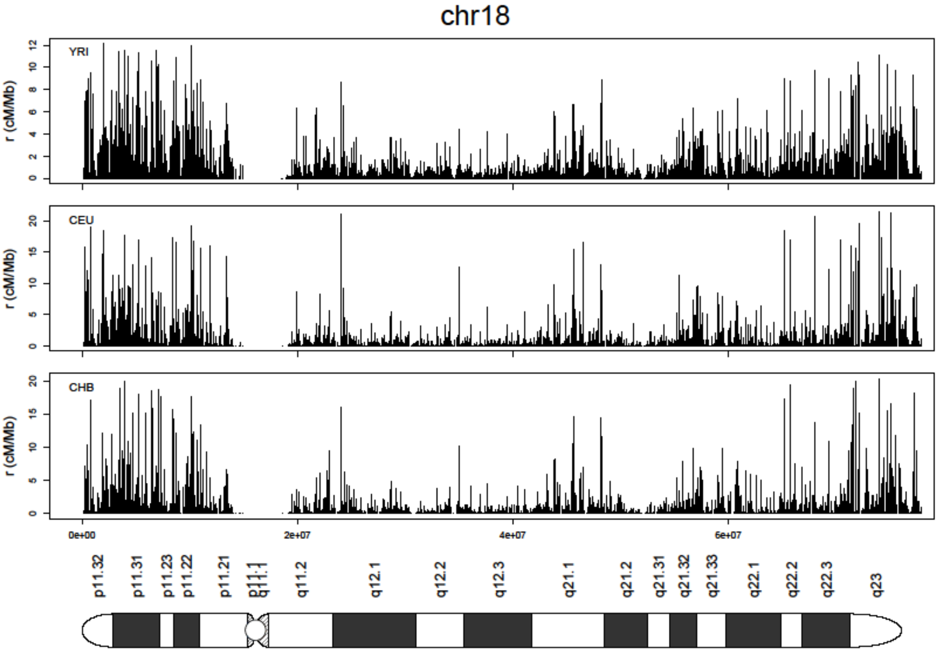
chr18
YRI
CEU
CHB
r (cM/Mb)
0e+00
2e+07
4e+07
6e+07
p11.32
p11.31
p11.23
p11.22
p11.21
q11.2
q12.1
q12.2
q12.3
q21.1
q21.2
q21.31
q21.32
q21.33
q22.1
q22.2
q22.3
q23

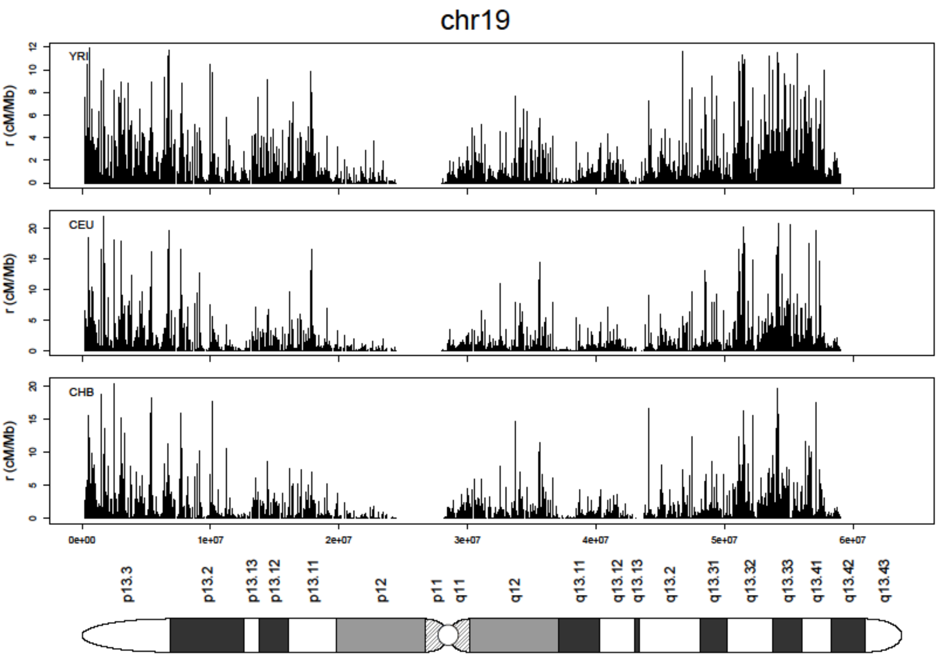

chr19
YRI
CEU
CHB
r (cM/Mb)
0e+00
1e+07
2e+07
3e+07
4e+07
5e+07
6e+07
p13.3
p13.2
p13.13
p13.12
p13.11
p12
p11
q11
q12
q13.11
q13.12
q13.13
q13.2
q13.31
q13.32
q13.33
q13.41
q13.42
q13.43

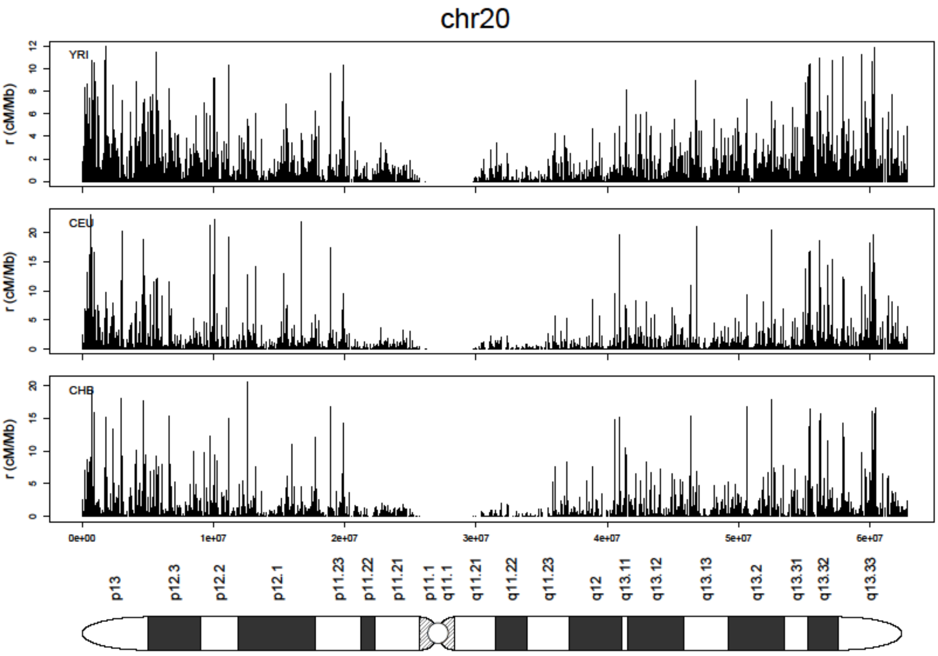

chr20
YRI
CEU
CHB
r (cM/Mb)
0e+00
1e+07
2e+07
3e+07
4e+07
5e+07
6e+07
p13
p12.3
p12.2
p12.1
p11.23
p11.22
p11.21
p11.1
q11.1
q11.21
q11.22
q11.23
q12
q13.11
q13.12
q13.13
q13.2
q13.31
q13.32
q13.33

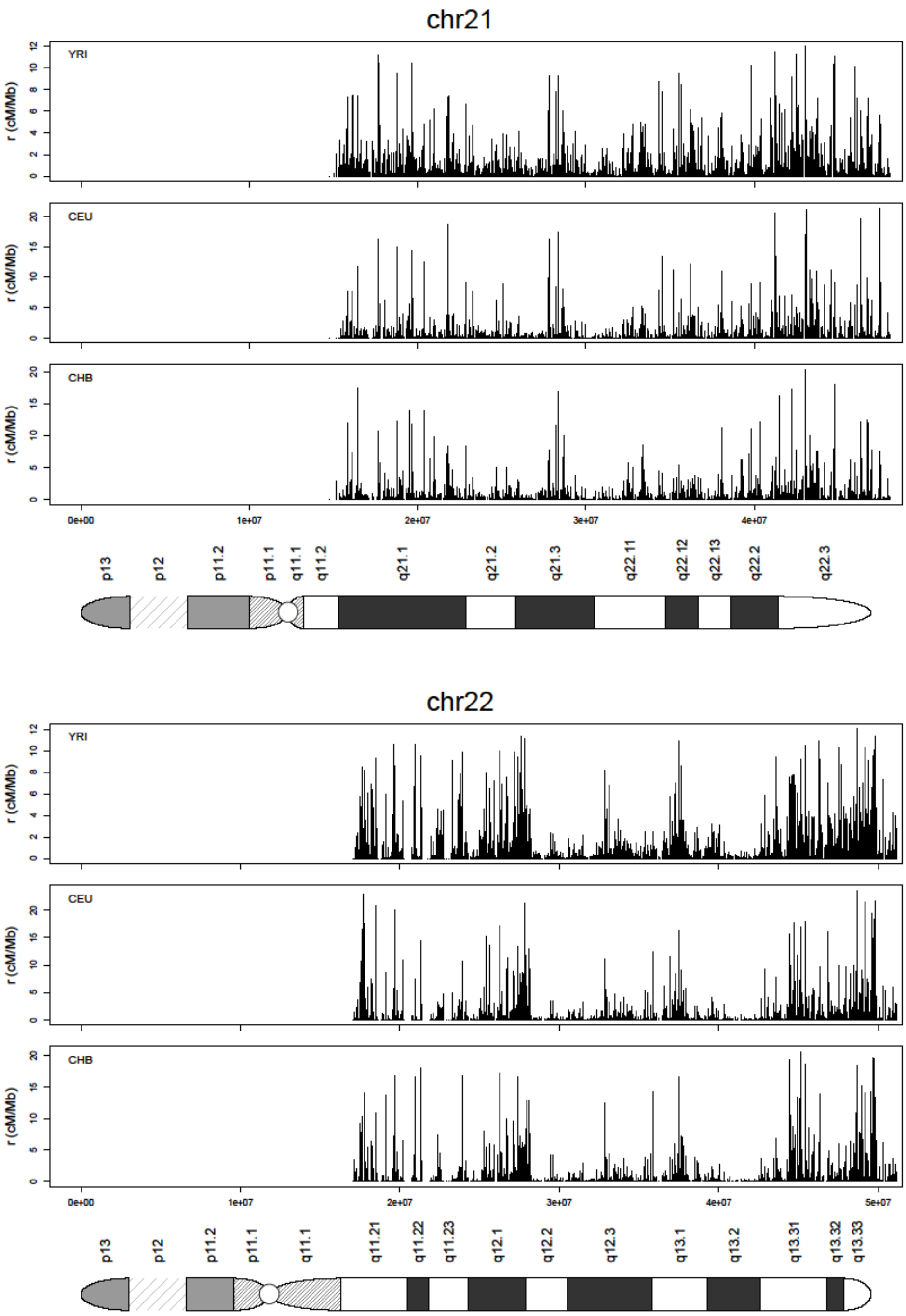

Figure S5 Recombination rates of the 22 autosomes for three human populations of African (YRI), European (CEU) and East Asian (CHB) ancestry at a 50-kb scale. The cartoon at the bottom is a visualization of the chromosome.
