# Supplementary material for: New Software for the Fast Estimation of Population Recombination Rates (FastEPRR) in the Genomic Era
Source: G3 (Bethesda). 2016 Mar 29;6(6):1563–71. doi: 10.1534/g3.116.028233 (PMC4889653; doi:10.1534/g3.116.028233)
Supplement: Supplemental Material [file supp_g3.116.028233_FigureS6.pdf]

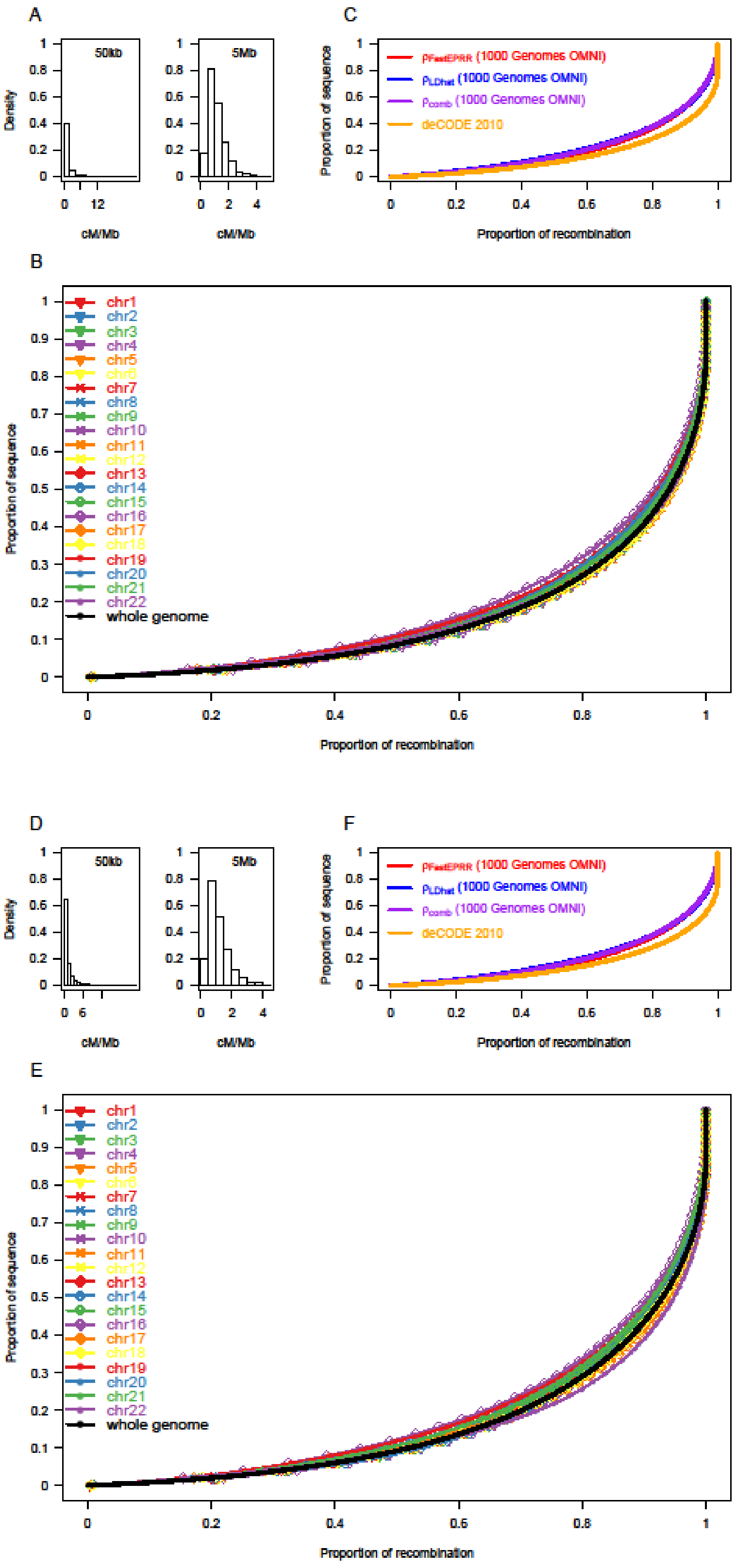

Figure S6 Recombination rate in European (CEU) (A, B, C) and East Asian (CHB) (D, E, F).

Histograms of the recombination rate for whole autosomal genome at 50-kb scale and 5-Mb

scales (A, D). Proportion of recombination in different fractions of sequence. Each colored line represents one chromosome and the black line denotes the whole autosomal genome (B, E). Concentration of recombination in a small proportion for the four genetic maps (C, F).
