# Supplementary material for: New Software for the Fast Estimation of Population Recombination Rates (FastEPRR) in the Genomic Era
Source: G3 (Bethesda). 2016 Mar 29;6(6):1563–71. doi: 10.1534/g3.116.028233 (PMC4889653; doi:10.1534/g3.116.028233)
Supplement: Supplemental Material [file supp_g3.116.028233_FigureS7.pdf]

(A) YRI 50-kb

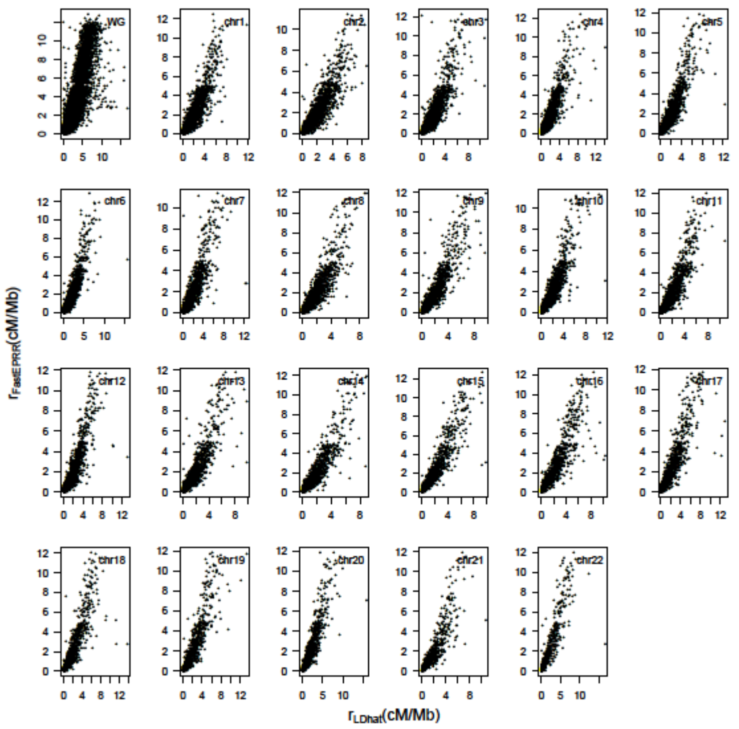

(B) YRI 5-Mb

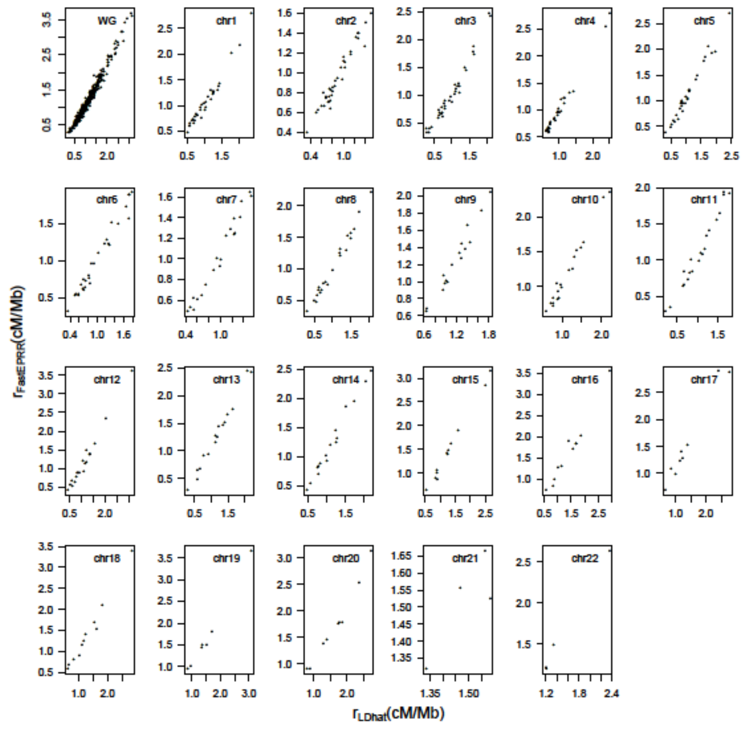

(C) CEU 50-kb

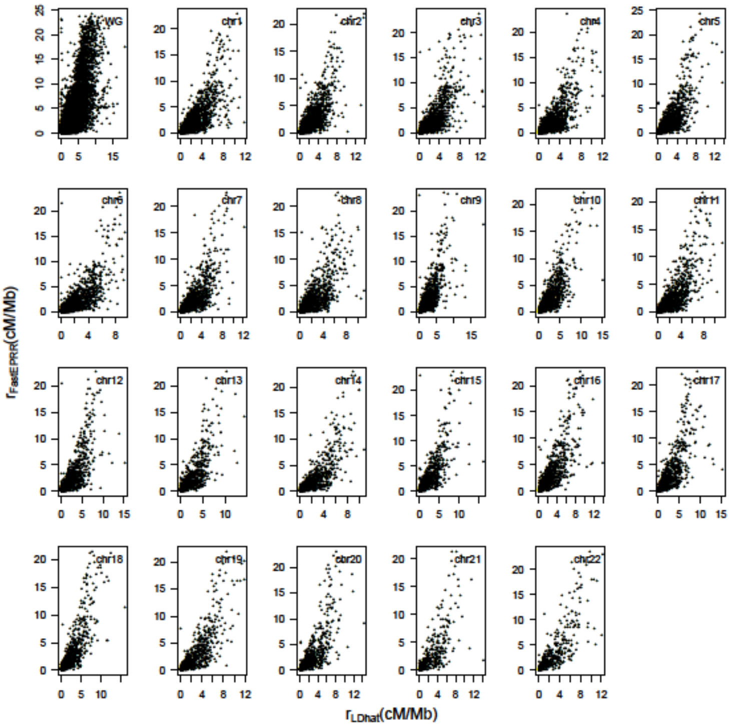

(D) CEU 5-Mb

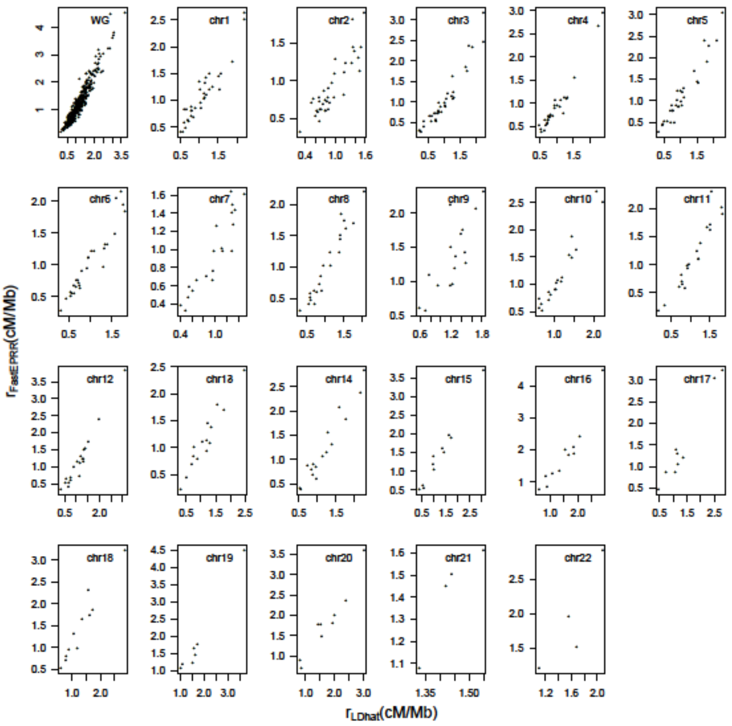

(E) CHB 50-kb

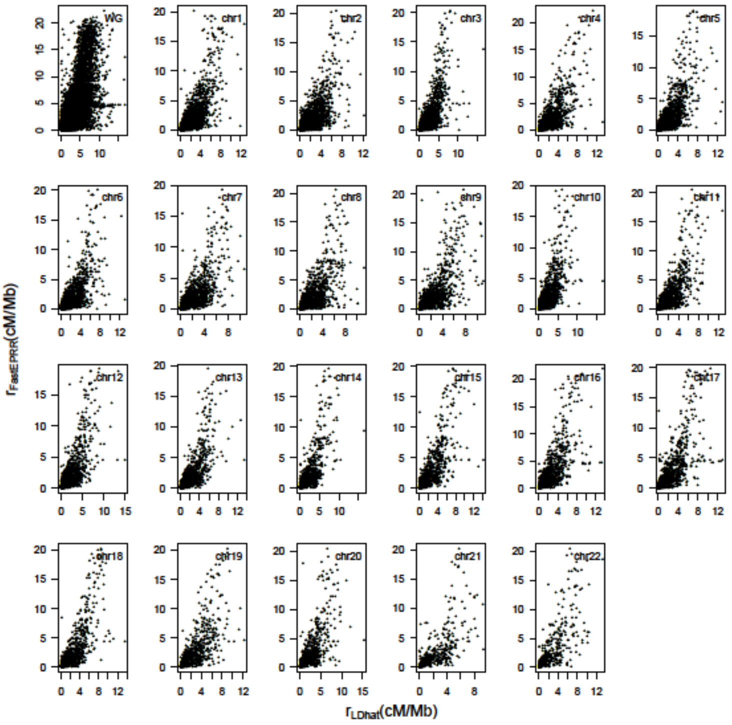

(F) CHB 5-Mb

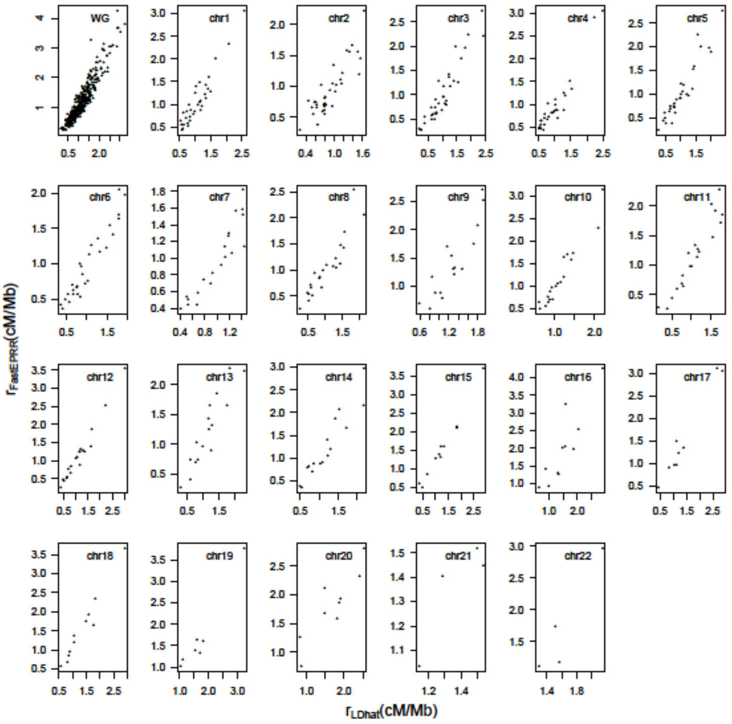

Figure S7 Comparisons of $\rho_{FastEPRR}$ and ρ_{LDhat} maps at 50-kb and 5-Mb scales for African (YRI) (A, B), European (CEU) (C, D) and East Asian (CHB) (E, F), respectively. The scatter plots show the comparison of the whole genome and 22 autosomes.
