# Supplementary material for: New Software for the Fast Estimation of Population Recombination Rates (FastEPRR) in the Genomic Era
Source: G3 (Bethesda). 2016 Mar 29;6(6):1563–71. doi: 10.1534/g3.116.028233 (PMC4889653; doi:10.1534/g3.116.028233)
Supplement: Supplemental Material [file supp_g3.116.028233_FigureS8.pdf]

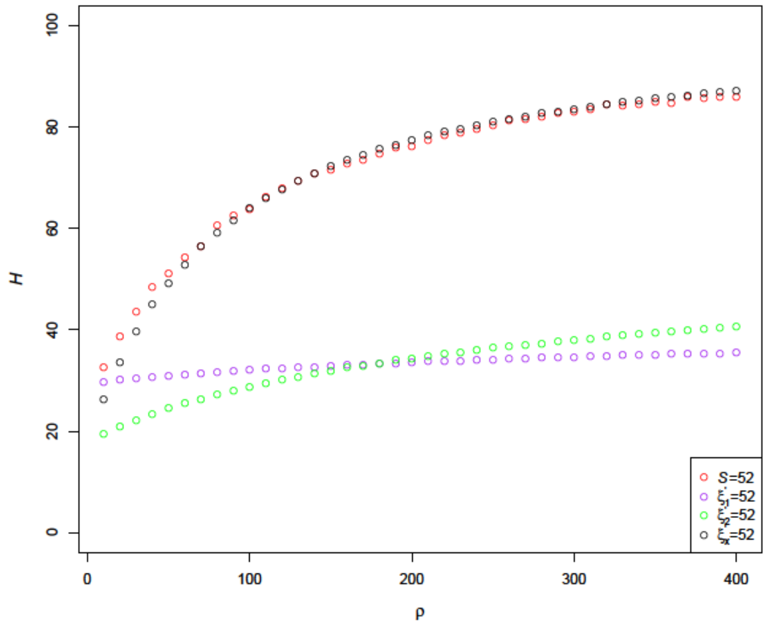


**Figure S8** The relationship between $H$ and $\rho$ under the condition of $\xi'_1, \xi'_2, \xi'_x$ and $S$ separately. $n = 100, S = 52, \xi'_1 = 52, \xi'_2 = 52, \xi'_x = 52$. The open circles represent the mean of all $H$ for each $\rho \in [10, 400]$.
