# Supplementary material for: New Software for the Fast Estimation of Population Recombination Rates (FastEPRR) in the Genomic Era
Source: G3 (Bethesda). 2016 Mar 29;6(6):1563–71. doi: 10.1534/g3.116.028233 (PMC4889653; doi:10.1534/g3.116.028233)
Supplement: Supplemental Material [file supp_g3.116.028233_FigureS9.pdf]

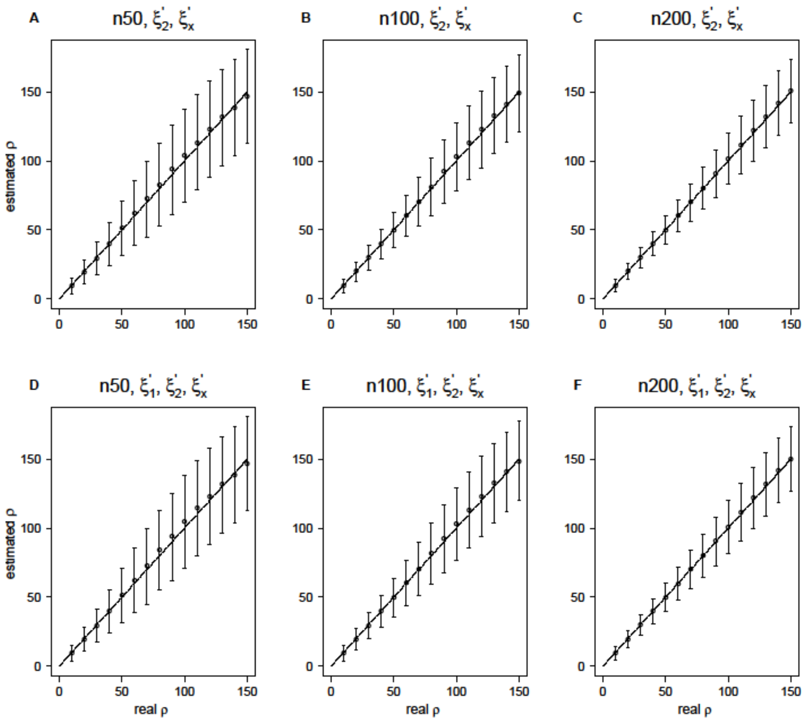

Figure S9 Comparisons of $\rho_{FastEPRR}$ with and without ξ_1'. $\hat{\rho}$ was estimated without ξ_1' (A-C) and with ξ_1' (D-F) for the sample sizes $n = 50$ (A, D), $n = 100$ (B, E) and $n = 200$ (C, F). The number of segregating site $S = 45$ $(n = 50)$, 52 $(n = 100)$ and 59 $(n = 200)$.
