# Supplementary material for: New Software for the Fast Estimation of Population Recombination Rates (FastEPRR) in the Genomic Era
Source: G3 (Bethesda). 2016 Mar 29;6(6):1563–71. doi: 10.1534/g3.116.028233 (PMC4889653; doi:10.1534/g3.116.028233)
Supplement: Supplemental Material [file supp_g3.116.028233_TableS1.pdf]

Table S1 RMSE of $\rho_{FastEPRR}$ when the sample size increases

n	RMSE	n	RMSE
50	17.6	500	8.2
100	12.3	600	8.0
200	9.8	700	8.0
300	8.7	800	7.8
400	8.4	1000	7.7

The RMSE of $\hat{\rho}$ was estimated from 2,000 simulated data conditional on real $\rho = 50$ and $S = 75$.
