# Supplementary material for: New Software for the Fast Estimation of Population Recombination Rates (FastEPRR) in the Genomic Era
Source: G3 (Bethesda). 2016 Mar 29;6(6):1563–71. doi: 10.1534/g3.116.028233 (PMC4889653; doi:10.1534/g3.116.028233)
Supplement: Supplemental Material [file supp_g3.116.028233_TableS2.pdf]

Table S2 Comparison of $\rho_{FastEPRR}$, ρ_{gam}, ρ_{LDhat} and ρ_{comb} for each sample size measured by RMSE

Real	$n = 50$				$n = 100$				$n = 200$			
ρ	FastEPRR	gam	LDhat	combined	FastEPRR	gam	LDhat	combined	FastEPRR	gam	LDhat	combined
10	5.9	15.5	5.4	4.0	5.0	11.8	4.5	3.3	4.4	9.0	4.3	3.0
20	8.5	13.6	8.3	5.9	6.8	7.2	7.3	5.0	5.8	4.5	6.5	4.3
30	11.1	20.1	11.4	8.0	8.5	11.4	9.9	6.6	7.2	8.5	8.4	5.5
40	15.0	25.8	15.2	10.7	10.6	15.0	12.5	8.2	8.6	11.2	10.4	6.7
50	18.6	30.4	18.4	13.1	12.7	17.4	14.6	9.7	10.0	12.6	12.4	7.9
60	22.1	32.6	21.6	15.6	15.0	19.1	17.4	11.5	11.6	13.5	14.2	9.2
70	25.7	33.6	24.6	18.0	17.7	21.2	19.4	13.0	13.1	14.8	16.4	10.4
80	27.9	33.2	27.4	19.7	20.3	22.8	21.8	15.0	14.9	16.5	18.1	11.7
90	29.9	33.2	30.3	21.5	22.5	24.2	24.1	16.6	16.9	18.5	20.2	13.2
100	31.6	29.9	33.2	23.2	25.0	24.6	25.7	17.9	18.8	20.4	22.2	14.6
110	32.3	27.4	35.4	24.0	26.0	24.6	27.9	19.1	20.4	21.0	24.0	15.8
120	33.1	24.7	36.7	24.8	27.1	24.2	29.3	20.0	21.9	21.5	25.8	17.0
130	33.5	22.7	37.6	25.0	27.8	22.7	30.7	20.7	22.7	21.0	27.6	18.0
140	33.7	22.0	37.7	25.5	28.0	21.5	31.7	21.0	22.7	19.7	28.9	18.5
150	34.3	22.9	37.9	26.0	27.7	20.9	32.0	21.2	22.7	18.3	29.2	18.5

The parameters in Figure 1 were used, unless noted otherwise.
