# Supplementary material for: New Software for the Fast Estimation of Population Recombination Rates (FastEPRR) in the Genomic Era
Source: G3 (Bethesda). 2016 Mar 29;6(6):1563–71. doi: 10.1534/g3.116.028233 (PMC4889653; doi:10.1534/g3.116.028233)
Supplement: Supplemental Material [file supp_g3.116.028233_TableS3.pdf]

Table S3 Computing time to analyze the genome-wide polymorphism data for three human populations

Population	Scanning (h)	Estimating (h)	Merging (h)	Total (h)
YRI	9.0	56.7	0.6	66.3
CEU	8.6	36.5	0.6	45.7
CHB	8.7	39.8	0.5	49.0

The unit of measurement is given in hours (h). A single CPU core of a computer with an AMD Opteron(tm) 800MHz processor was used. As expected, the waiting/computing time will be dramatically shortened when a computer cluster is available.
